# MicroRNA sequencing of rat hippocampus and human biofluids identifies acute, chronic, focal and diffuse traumatic brain injuries

**DOI:** 10.1038/s41598-020-60133-z

**Published:** 2020-02-24

**Authors:** Harris A. Weisz, Deborah Kennedy, Steven Widen, Heidi Spratt, Stacy L. Sell, Christine Bailey, Melinda Sheffield-Moore, Douglas S. DeWitt, Donald S. Prough, Harvey Levin, Claudia Robertson, Helen L. Hellmich

**Affiliations:** 1 The University of Texas Medical Branch at Galveston, Galveston, TX USA; 2 Baylor College of Medicine, Houston, TX USA

**Keywords:** Molecular neuroscience, Translational research

## Abstract

High-throughput sequencing technologies could improve diagnosis and classification of TBI subgroups. Because recent studies showed that circulating microRNAs (miRNAs) may serve as noninvasive markers of TBI, we performed miRNA-seq to study TBI-induced changes in rat hippocampal miRNAs up to one year post-injury. We used miRNA PCR arrays to interrogate differences in serum miRNAs using two rat models of TBI (controlled cortical impact [CCI] and fluid percussion injury [FPI]). The translational potential of our results was evaluated by miRNA-seq analysis of human control and TBI (acute and chronic) serum samples. Bioinformatic analyses were performed using Ingenuity Pathway Analysis, miRDB, and Qlucore Omics Explorer. Rat miRNA profiles identified TBI across all acute and chronic intervals. Rat CCI and FPI displayed distinct serum miRNA profiles. Human miRNA profiles identified TBI across all acute and chronic time points and, at 24 hours, discriminated between focal and diffuse injuries. In both species, predicted gene targets of differentially expressed miRNAs are involved in neuroplasticity, immune function and neurorestoration. Chronically dysregulated miRNAs (miR-451a, miR-30d-5p, miR-145-5p, miR-204-5p) are linked to psychiatric and neurodegenerative disorders. These data suggest that circulating miRNAs in biofluids can be used as “molecular fingerprints” to identify acute, chronic, focal or diffuse TBI and potentially, presence of neurodegenerative sequelae.

## Introduction

Among the global health challenges posed by traumatic brain injuries (TBI)^1^ is the need for better understanding of the TBI-induced mechanisms that lead to lasting and life-altering cognitive and neurobehavioral dysfunction and a need for improved classification of TBI subgroups. One potential approach is a “liquid biopsy,” a blood test such as that recently reported to permit earlier detection of eight common types of cancer^2^. Because small, non-coding microRNAs (miRNAs) are stable in circulating biofluids and have been identified as potential, non-invasive disease-specific biomarkers^3^, we hypothesized that circulating miRNAs may identify the occurrence of TBIs in acute and long-term survivors and that distinct miRNA profiles could potentially discriminate among subgroups of TBI patients.

MiRNAs post-transcriptionally regulate gene expression by translationally repressing mRNA targets; they regulate most cell signaling pathways involved in development, cell function and disease^4^. In the brain, altered miRNA expression is linked to neurodegenerative disorders^5^. In previous rat TBI studies, we reported that 24 h after experimental TBI, injury-induced miRNAs suppressed expression of pro-survival genes in dying hippocampal neurons^6^. These miRNA target genes, according to the Online Mendelian Inheritance in Man (OMIM) database, lead to human disease when dysregulated, mutated or deleted^7^. To test our hypothesis that persistent, long-term dysregulation of hippocampal miRNAs could partly explain chronic neurodegeneration after TBI, we designed a long-term survey of miRNA expression in rat hippocampus after TBI using unbiased next-generation sequencing (NGS).

Advances in NGS technologies have great potential to improve precision medicine and clinical practice^8^. Since our objective was to evaluate the translational potential of rat miRNA-seq results, we sequenced a limited set of archived human TBI serum samples representing multiple acute and chronic post-injury intervals and age-matched control serum. In these exploratory studies with rat and human tissues, we tested three hypotheses, 1) miRNA-seq profiles would identify TBI at all acute and chronic post-injury intervals, 2) miRNA-seq profiles would discriminate between focal and diffuse TBI and 3) serum miRNA-seq would reveal potential miRNA markers of neuropsychiatric and neurodegenerative sequelae.

## Methods

### Study design

**Figure 1:**
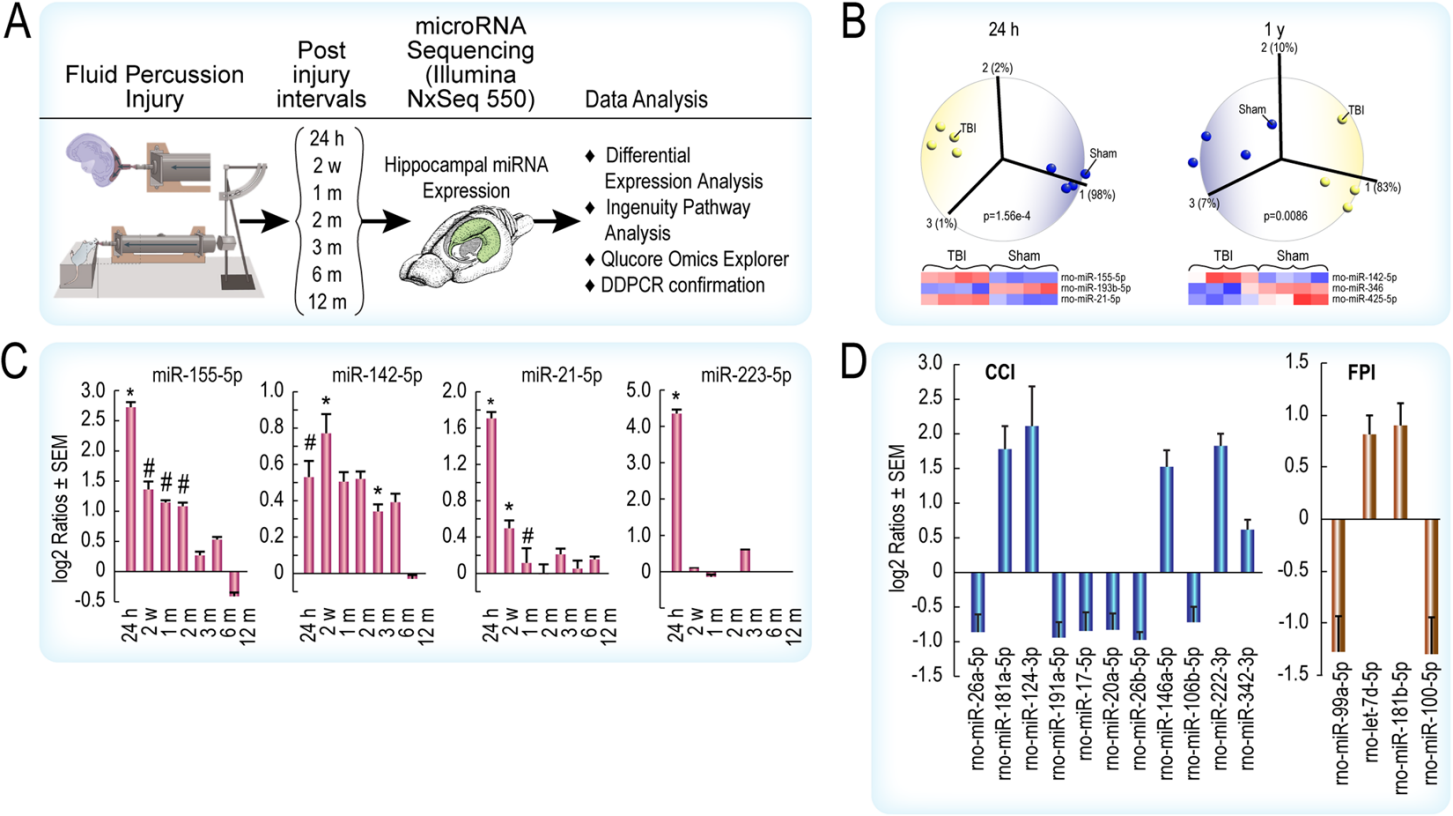
(**A**) Experimental workflow for miRNA-seq analysis of rat hippocampus after fluid percussion injury (FPI). (**B**) Principal component analysis (PCA) revealed miRNAs capable of discriminating between TBI and sham-injured controls at 24 h to 1 y post FPI. (**C**) Analysis of miRNA-seq data in EdgeR and DESeq2 (using both the Wald test and LRT [Likelihood-ratio test] in each) identified four miRNAs that were significantly different compared to sham injured controls in at least one post-injury interval. The *denotes significant [FDR < 0.05] differential expression in both Wald and LRT tests and #denotes significant [FDR < 0.05] differential expression per Wald or LRT test. (**D**) Differentially expressed rat serum miRNAs that are significant compared to sham-injured rats by PCR array analysis after FPI or CCI. Data are displayed graphically as the log2 ratio ± SEM along the y-axis and miRNAs are listed along the x-axis. Data were analyzed through the use of IPA (QIAGEN Inc., https://www.qiagenbioinformatics.com/products/ingenuitypathway-analysis) and Qlucore Omics Explorer.

**Figure 2:**
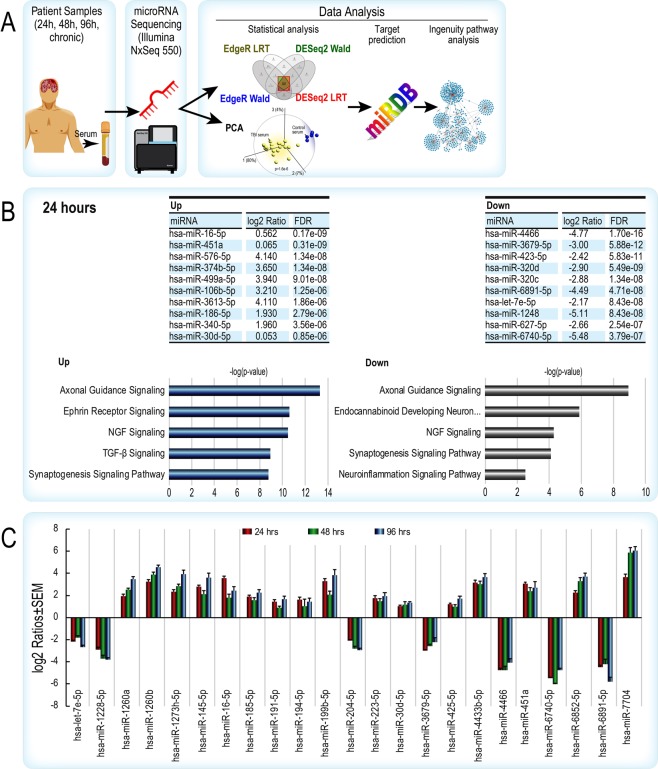
(**A**) Experimental workflow for miRNA-seq and bioinformatic analysis of human uninjured control and TBI patient samples. (**B**) The top 10 up- and down-regulated miRNAs by level of significance (FDR adjusted p-value) in the 24 hr patient cohort. Ingenuity Pathway Analysis of predicted gene targets show the top nervous system-related canonical pathways for the up and down miRNAs, respectively are involved in synaptic plasticity, neurorestorative signaling and neuroinflammation. The x-axes are based on the -log of the Fisher’s Exact p-value. (**C**) Consistent expression of miRNAs across three acute periods after TBI. Data is displayed graphically as the log2 ratio ± SEM (y-axis) and significant miRNAs from the LRT and Wald tests in EdgeR and DESeq2 (x-axis). Red bars indicate 24 hrs, green 48 hrs, and blue 96 hrs after TBI. SEM – standard error of the mean; LRT – likelihood ratio test; hsa – human miRNA. Data were analyzed through the use of IPA (QIAGEN Inc., https://www.qiagenbioinformatics.com/products/ingenuitypathway-analysis).

The miRNA-seq and data analyses workflow is shown in Fig. 1A (rat studies) and Fig. 2A (human studies). Detailed methods, including rat surgical procedures, are described in Supplementary Materials and are summarized below. All procedures were done under a protocol (No. 1312056A) approved by the University of Texas Medical Branch at Galveston’s Institutional Animal Care and Use Committee. All procedures performed as part of this study were performed in accordance with institutional, state, and U.S. federal guidelines and regulations.

In the rat studies, for each post-injury interval, we sequenced four TBI and four sham-control hippocampi, thus 56 rats were used for miRNA-seq analysis. In the human studies, we sequenced 51 serum samples (six acute and six aged controls, 33 acute TBI representing 24, 48, 96 h post-injury, six chronic TBI [2–32 years post-injury]). Demographic characteristics are shown in Table 1.

**Table 1:** Patient Demographics and Characteristics.

Variables	24 h*	48 h*	96 h*	Acute	Chronic	Chronic
	(n = 21)	(n = 7)	(n = 5)	Controls (n = 6)	TBI (n = 6)	Controls (n = 6)
Age in years, mean (SD)	34.19 (14.5)	37.86 (14.70)	35.40 (16.53)	32.67 (5.29)	56.00 (9.42)	58.17 (2.80)
GCS, avg.	7.38	6.00	5.60	N/A	ND	N/A
IMPACT (Risk of death)	24.10	29.14	26.00	N/A	ND	N/A
IMPACT (Risk of poor outcome)	40.33	45.14	40.40	N/A	ND	N/A
Sex (M or F)	16M, 5F	6M, 1F	5M	3M, 3F	ND	3M, 3F
Injury Subtype: Focal	15	5	3	N/A	ND	N/A
Injury Subtype: Diffuse	6	2	2	N/A	ND	N/A

GCS: Glasgow Coma Score.

IMPACT: International Mission for Prognosis and Analysis of Clinical Trials (percent risk).

*Blood samples obtained 24, 48 and 96 h post-TBI. 48 h and 96 h blood samples were obtained from a subset of the 24 h TBI.

### Rat hippocampus and serum isolation and PCR array analysis

Total RNA from rat hippocampal tissue was extracted and purified using Ribopure (Ambion) as in Boone and Weisz *et al*.^9^. Serum from rats subjected to FPI and CCI was used for miRNA isolation and PCR array analysis as described in Supplementary Methods.

### Human serum miRNA isolation and precipitation

Archived human TBI serum was used for this pilot study. The samples were initially collected as part of a clinical trial of erythropoietin^10^, and informed consent was obtained from all subjects or their legally authorized representatives for the samples remaining after analyses performed for the trial were completed to be banked and used in future studies. The de-identified samples were stored and distributed under IRB protocol H-13606 at Baylor College of Medicine (Houston, TX; C.R., PI). Use of these samples in the present study was deemed as exempt from review as a human subjects protocol by the IRB at the University of Texas Medical Branch at Galveston, as the materials were from an established tissue/sample bank, had been de-identified, etc. All procedures performed as part of this study were performed in accordance with institutional, state, and U.S. federal guidelines and regulations.

The samples that were selected were from six patients who had an injury that was predominantly a diffuse injury pattern with either a normal CT scan or scattered shear injuries, and 15 patients that had a focal contusion. Detailed miRNA isolation procedures are provided in Supplementary Methods.

### Small RNA sequencing

Small RNA libraries were made using the NEBNext small RNA Multiplex kit (New England Biolabs, Inc.). The library preparation method included a final size fractionation to enrich for inserts of ~22 bases. After purification by polyacrylamide gel-electrophoresis, the sample libraries were pooled and sequenced by the UTMB Next Generation Sequencing Core on an Illumina NextSeq550 (single end 75 base) using TruSeq SBS kit v3 (Illumina) and protocols defined by the manufacturer. The miRDeep2 software package, version 2.0.0.8, was used to trim adapter sequences from the reads and quantify miRNA read counts using the miRBase database, release 22. On average, about 13 million reads were obtained per sample, approximately 80% of the reads passed trimming and filtering and approximately 1.3 million reads per sample mapped to miRNAs. The rat and human miRNA-seq data has been deposited in the National Center for Biotechnology Information’s Gene Expression Omnibus (GEO Accession numbers GSE131695 and GSE131704).

### MicroRNA target prediction and ingenuity pathway analysis

To infer the regulatory actions of miRNA from sequencing data and improve our confidence that these miRNAs are representative of brain pathology, we performed an iterative process for miRNA target prediction and pathway analysis using a miRNA target prediction online suite, miRDB and Ingenuity Pathway Analysis software; details provided in Supplementary methods.

### Statistical analysis

Differential expression analysis of the miRNA-seq counts was done using two R packages: Empirical Analysis of Digital Gene Expression Data in R (EdgeR) and Differential gene expression analysis based on the negative binomial distribution (DESeq2). Both use the negative binomial distribution to assess differences in miRNA expression using count data and a general linear model (glm) analysis framework. The glm procedure was used to estimate two exact tests: the quasi-likelihood (QL) F-test (the Wald Test) and the likelihood ratio test (LR test). The top tags from each were read out and compared. Additional details are provided in Supplementary Methods.

### Principal component analysis

Qlucore Omics Explorer (QOE, Qlucore, Lund, Sweden) is an intuitive data analysis and data mining software tool that combines speed and advanced analytics for interactive exploration and instant visualization of multivariate data; QOE is built on state-of-the-art mathematical and statistical methods (a general linear statistical model based on R). The dynamic visualization tool in QOE is principal component analysis (PCA), an unsupervised (agnostic to variable and sample annotations), descriptive and data reduction technique^11^ which is derived from calculation of the covariance among the original variables in the data. In essence, PCA extracts essential information from high-dimensional data by finding the directions of maximum variance (how far each value in the dataset is from the mean) and then captures or projects the variance in these many variables into a smaller, easier-to-analyze set of variables. The interactive, PCA approach facilitates heuristic data analysis and enables visualization of the miRNA data in a three-dimensional space after filtering out variables with low overall variance and statistical testing. Because PCA is used both for exploratory data analysis and as a machine learning tool for predictive models, this unsupervised statistical technique is suitable for finding patterns in high-dimensional data such as miRNA-seq.

### Funding

This study was supported by the Moody Project for Translational Traumatic Brain Injury Research. The funders had no role in the study design, data curation, data analysis, data interpretation, writing of the report or decision to submit for publication.

## Results

### Rat studies

We examined FPI-induced miRNA changes in rat hippocampus (Fig. 1A) at seven post-injury intervals (24 h, 2 w, 1, 2, 3, 6 and 12 m). Principal component analysis distinguished TBI from sham controls at all seven post-injury intervals (24 h PCA and one y PCA shown in Fig. 1B, all other time points in Fig. S1A). Four miRNAs (miR-155-5p, miR-142-5p, miR-21-5p and miR-223-5p) that were differentially modulated across multiple post-injury intervals (droplet digital PCR confirmation of miRNA expression shown in Fig. S1B), are prominently linked to immunoregulatory functions (Fig. 1C, Table S1, Supplementary References). For instance, examination of TLR (toll-like-receptor)-mediated NFκB signaling shows that multiple differentially expressed miRNAs regulate inflammatory gene expression (Fig. S1C). These observations are concordant with our previous observations of chronic dysregulation of immune response genes in the TBI rat brain^9^.

To test our hypothesis that circulating miRNAs may reflect TBI-induced changes induced by focal or diffuse injuries, we performed serum miRNA PCR array analysis of rats subjected to CCI (predominantly focal) or FPI (mixed diffuse/focal). CCI and FPI produced distinct miRNA expression profiles (Fig. 1D).

### Human studies

#### Acute Injury (24–96 hours post injury)

We performed and analyzed miRNA-seq data (Fig. 2A) from archived human serum samples obtained at three post-injury intervals (24, 48 and 96 h). Demographic information for TBI patients and uninjured controls is provided in Table 1. Diagnosis of focal or diffuse TBI was based on CT imaging. Principal component analysis and hierarchical clustering clearly distinguished all TBI patients from healthy, age-matched controls (Fig. S2A–C). We identified a common set (from four statistical tests in R) of differentially expressed serum miRNAs for each individual post-injury time point (24 h in Fig. 2B, 48 and 96 h in Fig. S3). Ingenuity pathway analysis of predicted gene targets of significantly dysregulated miRNAs indicated a prominent representation of CNS pathways involved in neural plasticity, neuro-restorative processes, synaptic communication and neuroinflammation (Figs. 2B, S3A,B). Bioinformatic and pathway analysis of predicted gene targets of the top ten up- and down-regulated miRNAs in all TBI groups indicated that the same CNS pathways were impacted by TBI across all acute post-injury intervals (Figs. 2B, S3A,B). Note that the analyses of miRNAs and pathways are shown this way for clarity of presentation. We found that 23 miRNAs that target the top CNS pathways identified by IPA were similarly increased or decreased across all acute time points (Fig. 2C). With additional validation with more patient samples, a combination of these may serve as legitimate circulating biomarkers of acute TBI.

#### Focal vs diffuse injury

**Figure 3:**
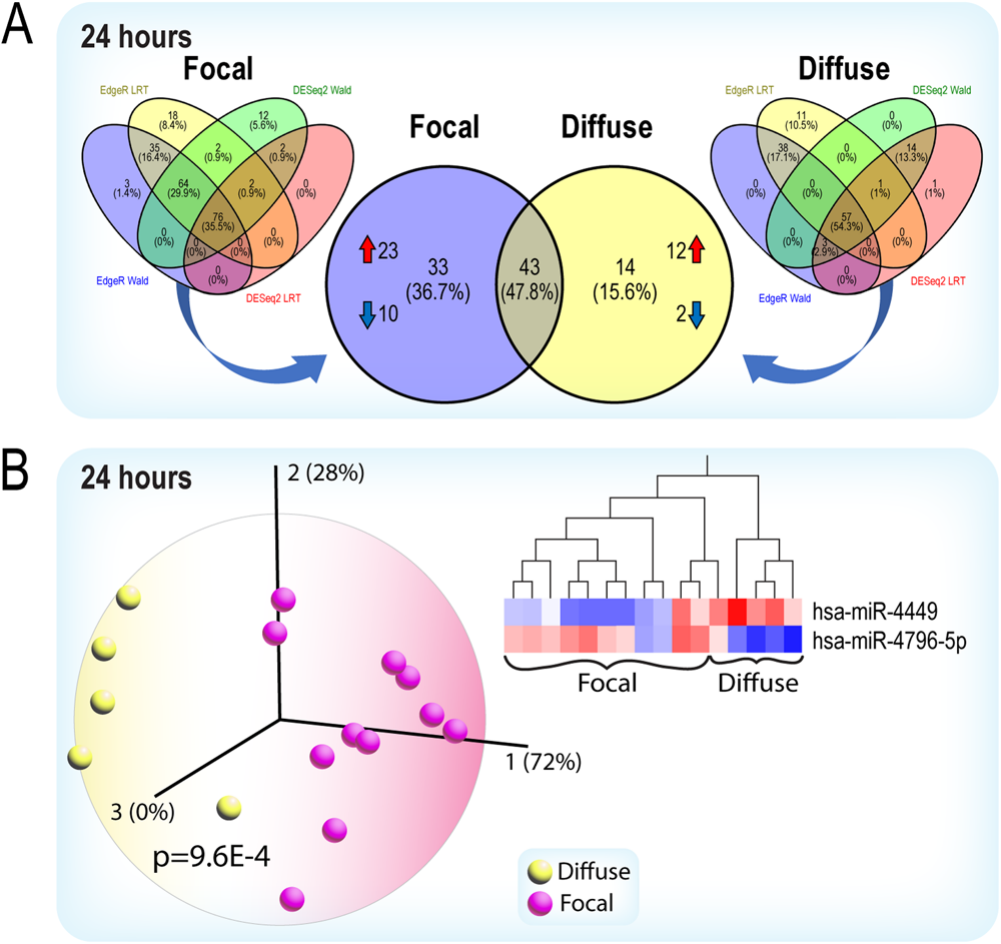
(**A**) Patients in the 24 h cohort were separated based on injury designation, into either focal or diffuse injury categories. Overlapping significant miRNAs from the LRT and Wald tests in EdgeR and DESeq2 (compared to healthy controls) in each respective group are shown in the left and right Venn diagrams. Thirty-three miRNAs (23 up and 10 down) are unique to the focal injury group (purple circle, center Venn diagram). Fourteen (12 up and 2 down) are unique to the diffusely injured group (yellow circle, center Venn diagram). (**B**) Principal component analysis and the hierarchical clustering heatmap shows that two miRNAs discriminate the focal from the diffuse injury groups. The first two principal components represent 100% of the variance in the miRNA-seq data. Data were analyzed through the use of IPA (QIAGEN Inc., https://www.qiagenbioinformatics.com/products/ingenuitypathway-analysis) and Qlucore Omics Explorer.

**Figure 4:**
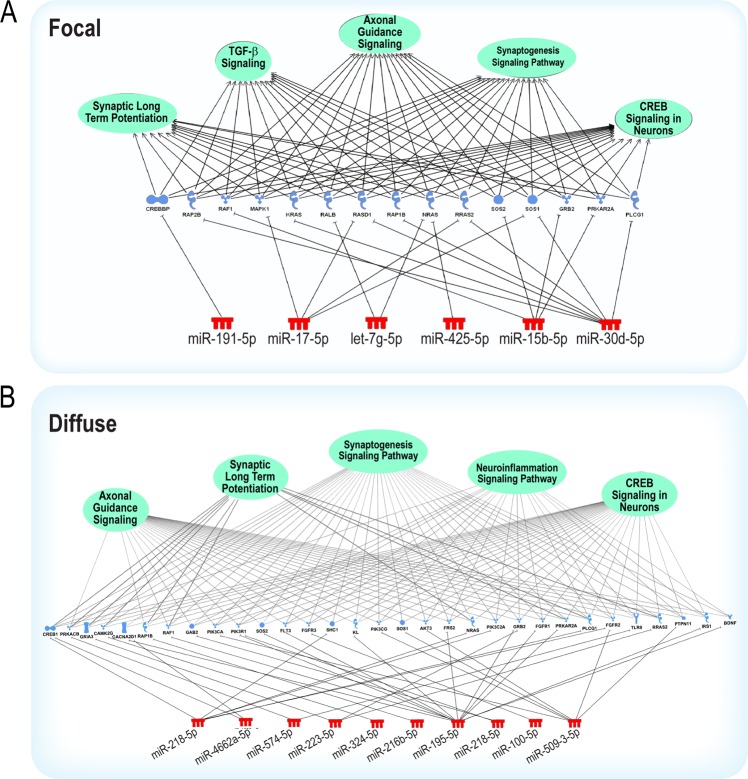
Pathway analysis reveals predicted gene targets enriched in predominately nervous system signaling pathways for focal (**A**) or diffuse (**B**) injury cohorts. miRNA colors signify increased (red) expression compared to control samples. The color of the gene targets represents the predicted direction of regulation based on target-prediction using miRDB. The green circles represent biological pathways comprising of predicted genes based on Ingenuity Pathway Analysis. Data were analyzed through the use of IPA (QIAGEN Inc., https://www.qiagenbioinformatics.com/products/ingenuitypathway-analysis).

Due to sample limitations, statistical and bioinformatic analysis of diffuse and focal injuries were only performed for the 24 h group. Despite similar GCS and ISS (Avg GCS, diffuse 6.5, focal 7.3, Avg injury severity scores, diffuse 36, focal 30), we found that serum miRNA profiles clearly discriminate between focal and diffuse TBIs (Fig. 3A). Each injury subtype is represented by a distinct panel of up or downregulated miRNAs (Fig. S4, focal S4A, diffuse S4B). The Venn analysis (Fig. 3A) illustrates that different statistical algorithms yield different lists of “significant” miRNA variables. Therefore, we focused on miRNAs that are common to all four statistical analysis tools. Principal component analysis and hierarchical clustering clearly distinguish focal and diffuse TBIs (Fig. 3B). Pathway analysis of the predicted gene targets show that CNS signaling pathways are prominently affected by both injury subtypes (Fig. S4), but interestingly, there is a prominent representation of pathways involved in immunoregulatory functions (e.g. AMPK, PTEN, B Cell Receptor signaling)^12^ in diffuse injuries (Fig. S4B). However, the overlapping, complex regulation by differentially expressed miRNAs (Figs. 4, S5 and S6) implicated in focal and diffuse injuries illustrates the difficulties in differentiating between TBI subtypes based solely on a molecular analysis.

#### Chronic injury (2–32 years post injury)

**Figure 5:**
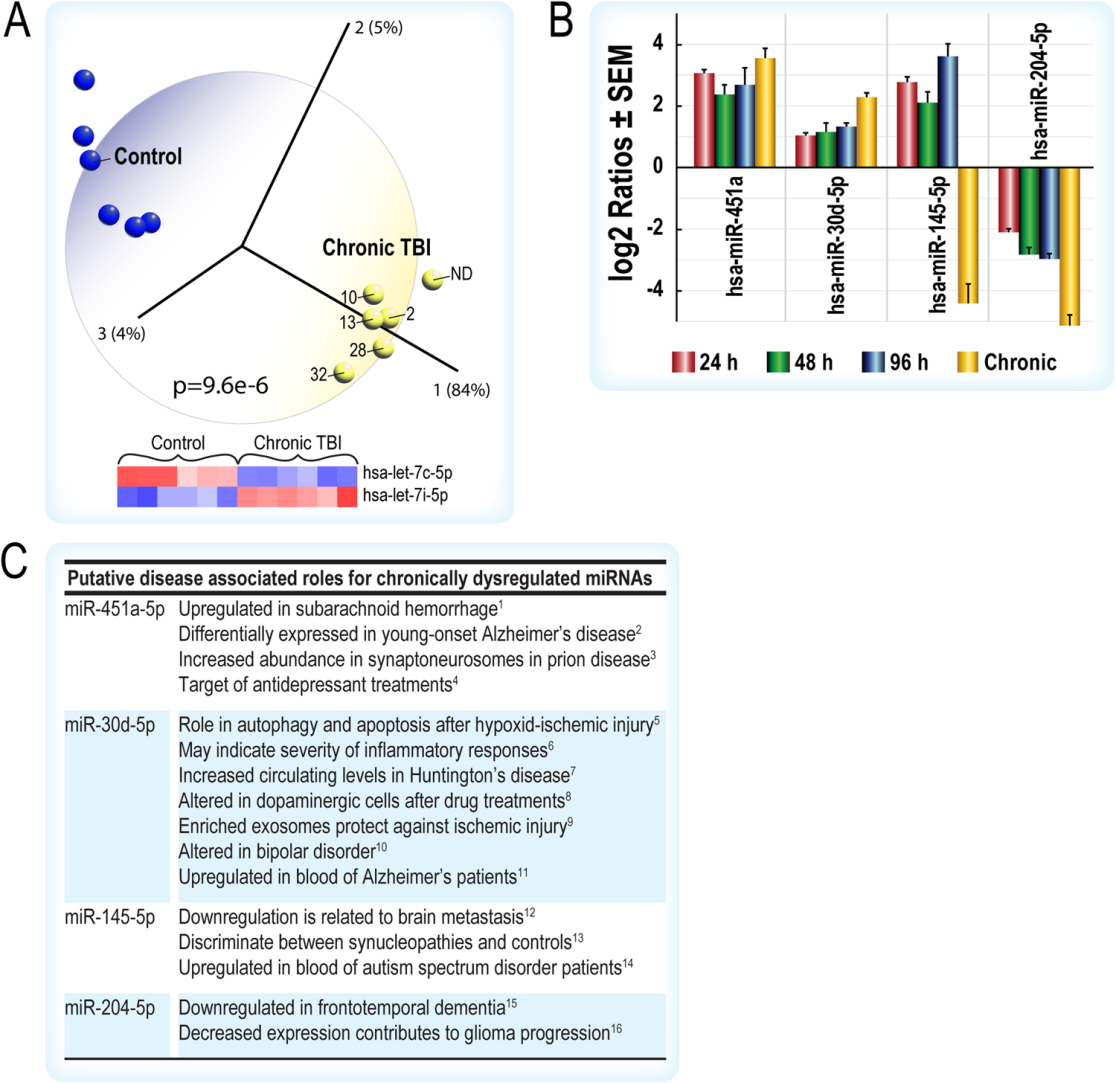
(**A**) Principal component analysis and the hierarchical clustering heatmap shows that two members of the let-7 family (hsa-let-7c-5p and has-let-7i-5p) clearly discriminates the chronic TBI cohort (2,10,13,28,32 years post-injury, one individual had no data [ND] but was classified as chronic TBI) from healthy, age-matched controls. (**B**) Four acutely up-regulated miRNAs remain dysregulated in chronic TBI patient serum. Data is displayed graphically as the log2 ratio ± SEM (y-axis) and significant miRNAs from the LRT and Wald tests in EdgeR and DESeq2 (x-axis). Red bars indicate 24 h, green 48 h, blue at 96 h post-TBI when compared to healthy controls. Orange bars indicate chronic TBI patient serum compared to healthy, age-matched controls. (**C**) miRNAs dysregulated in chronic TBI are associated with multiple brain disorders (citations in Supplemental references for Fig. 5C). Data were analyzed through the use of IPA (QIAGEN Inc., https://www.qiagenbioinformatics.com/products/ingenuitypathway-analysis) and Qlucore Omics Explorer.

We analyzed miRNA-seq data from a limited number of chronic human TBI serum samples (2 to 32 years post-TBI, one [ND] identified as chronic TBI only) and age-matched controls (Fig. 5). Principal component analysis and hierarchical clustering showed that all chronic TBIs can be distinguished from age-matched controls (Figs. 5A, S7) and that differential expression of two let-7 isoforms were sufficient to identify each group (Fig. 5A). Although few individual miRNAs were commonly dysregulated across the acute and chronic TBI groups, the primary observation was that the top IPA pathways targeted by these miRNAs are similar across all acute and chronic intervals (Figs. 2B, S3 and S8). *In silico* analysis showed that four miRNAs (Fig. 5B), differentially expressed across all acute and chronic post-injury intervals, are implicated in multiple human diseases, including neuropsychiatric (e.g., depression) and neurodegenerative (e.g., Alzheimer’s and Huntington’s) disorders (Fig. 5C).

#### Rat and human microRNA concordance

At the miRNA level, the rat and human results were not concordant. However, examination of commonly altered pathways populated by genes regulated by upregulated and downregulated miRNAs in rat and human TBI (Fig. S9) indicates persistent dysregulation of CNS pathways involved in neuroplasticity, cell-cell signaling and brain connectivity as the underlying neuropathological mechanisms of acute and chronic TBI. On the other hand, we noted the prominence of neuro-restorative signaling (i.e., axon guidance, CREB, neurotrophin signaling) in the gene targets of downregulated miRNAs.

## Discussion

This proof-of-concept study began with the goal of deciphering the underlying molecular progression of chronic TBI in the rat hippocampus using miRNA sequencing. With several insights gained from these data and from analyses of serum miRNA profiling data generated in two rat TBI models, we conducted a pilot study of human TBI using a limited number of archived acute and chronic TBI serum samples. From our survey of existing human TBI miRNA studies (see Supplemental discussion and references), we surmised that the key results of our pilot study have not been previously reported. Given the current interest in blood-based tests for human diseases^13^, bioinformatic and *in silico* analyses resulted in three key findings that may inform the potential of blood-based tests for TBI.

First, despite the relatively poor concordance of rat and human miRNA data, in both species, miRNA profiles clearly distinguish all cases of brain injury from uninjured, age-matched controls across all acute and chronic post-injury intervals. Notably, the unequivocal identification of human TBI survivors up to 32 years post-injury is not only clinically relevant but, at the molecular level, demonstrates that TBI is indeed a chronic disease process, not just a single event^14^. Moreover, a miRNA-based blood test would also serve as additional confirmation of chronic TBI obtained via neuroimaging^15^. This study also indicates that, using miRNA-seq profiling of peripheral biofluids, we would be able to study the continuum and progression of TBI at the molecular genetic level to identify therapeutic windows throughout the lifetime of TBI survivors.

Second, in both rats and humans, focal and diffuse brain injuries result in distinct serum miRNA profiles. Although a previous study showed differences in focal and diffuse injuries in living biopsies of human brain tissue^16^, we show here the first evidence that blood miRNA profiles could reflect the heterogeneity and injury-specific pathogenic changes induced in the brain by a diffuse or focal TBI which, at present, are only conclusively identifiable by sophisticated neuroimaging^15^. Given that genetic factors affect the heterogeneous presentation of TBI and functional outcome after TBI^17^, it is notable that discriminating focal from diffuse injuries by principal component analysis is congruent with the diagnosis by CT imaging. In future, we may be able to define minimally invasive circulating miRNA profiles that correspond to other specific subtypes of TBI such as hemorrhagic, penetrating and ischemic injuries.

Third, *in silico* analyses indicated that at longer times (two – 32 years) after TBI, differentially expressed miRNAs regulate critical CNS functions that are linked to well-known neuropsychiatric and neurodegenerative sequelae of TBI^18^. Given recent studies demonstrating that common neurobiological substrates (i.e. shared molecular genetic mechanisms) underlie different brain disorders^19^, these data suggest that some TBI dysregulated miRNAs may be causally implicated in the increased risk of, or may potentially serve as surrogate markers of, these brain disorders. For instance, changes in miR-199 and miR-34 have been linked to status epilepticus^20^. And chronically upregulated miR-1908-5p is predicted to regulate multiple genes involved in glutamatergic synaptic function^21^. Notably, miR-204-5p which was downregulated in chronic TBI was also reported downregulated in frontotemporal dementia^22^, a finding that suggests that this miRNA might serve as a useful biomarker of early stage, subclinical dementia in neurodegenerative disorders. TBI-dysregulated miRNAs also regulate multiple genes involved in neurodevelopmental functions^23^; Schork *et al*., showed in their study of shared risk across psychiatric disorders, that all share dysregulated expression of common gene variants that regulate neural development^24^. In fact, two isoforms of let-7, which are critically involved in brain development^25^, can identify chronic TBI patients two to 32 years post-injury. That these miRNAs are still dysregulated at 32 years after injury is evidence of long-lasting changes after a TBI, suggesting that psychological and cognitive impairment can persist for long times after TBI. Therefore, treatment of chronic TBI survivors is worthwhile.

A recent study of epigenetic dysregulation in a spectrum of Alzheimer’s Disease (AD) patients showed hypomethylation of AD-associated enhancer genes; hypomethylation of enhancers in prefrontal cortex neurons results in aberrant activation of cell cycle and upregulation of AD-associated genes such as BACE1^26^. Bioinformatic analyses indicated that these AD-associated genes are regulated by multiple TBI-associated miRNAs, notably miR-9 and miR-125, which are downregulated in chronic TBI; this suggests that these AD-associated genes are upregulated. Given evidence that TBI accelerates brain aging^27^, we found that at 24–96 h post-injury and as long as 32 years post-injury, miRNAs implicated in brain aging^28^ are still found to be dysregulated. One positive finding is that the persistent upregulation of some miRNAs, such as miR-451, in chronic TBI is a sign of a restorative response; for example, increased levels of miR-451 via diet has been shown to protect cells from oxidative stress^29^.

The rat hippocampal miRNA-seq data are largely congruent with the long-lasting immune dysregulation that we found in our recent study of chronic TBI-induced gene expression^6^ and suggests both positive and negative consequences of dysregulated miRNAs. Some fish, amphibians and reptiles have regenerative properties which are not found in humans^30^. A potential explanation is that in humans, injury-induced miRNAs such as miR-155 decreased expression of antioxidant genes such as catalase and superoxide dismutase^31^, thus inhibiting regenerative signaling^32^. Moreover, as both miR-155 and miR-223 have functional roles in mitochondria and cytoplasm^33^, their dysregulation for several months after TBI suggests persistent mitochondrial dysfunction. On the other hand, upregulation of miR-223 may reflect a protective, regenerative response. MiR-223 inhibits inflammasome activation through caspase-1 and IL-1β^34^. By targeting glutamate receptors, GluR2 and NR2B, miR-223 also inhibits calcium influx and protects neurons from excitotoxic damage^35^. We also inferred that upregulation of miR-21 may contribute to neurodegenerative signaling given its role in proliferation and differentiation of neural stem cells via regulation of AKT and GSK-3β^36^.

Recent studies show that the difficulties in translating rodent pathophysiological processes to human is because TBI-induced pathological changes occur on different timescales and there is a lack of a universal “conversion” rate^37^. However, we show that, despite the differences in individual miRNAs, there are many commonly affected gene targets in cellular pathways collectively affected by TBI-dysregulated miRNAs in both rat and human tissues. Thus, in lieu of focusing on individual genes or miRNAs, it may be more relevant to study the commonly modulated CNS pathways affected by TBI in both species.

In previous studies^6^, we showed in dying hippocampal neurons, miR-15b suppressed the expression of brain-derived neurotrophic factor (BDNF), a neurotrophic factor that is essential for brain survival and cognitive function^38^. Examination of the 3′UTR of the human BDNF gene shows multiple binding sites for human miRNAs chronically altered after injury, suggesting that dysregulated BDNF levels are involved in decline of cognitive functions after TBI. Given evidence of disrupted brain connectivity networks in brain diseases such as depression and Alzheimer’s^39^, our bioinformatic analysis indicated that miRNAs altered by acute and chronic TBI collectively regulate myriad gene networks involved in brain connectivity^40,41^.

We present this analysis with some caution since, in addition to the limited sample sizes in this exploratory pilot study, it is possible that some of the miRNA changes may not be specific to brain injury given that some of the TBI patients had some elements of polytrauma. Moreover, the samples were selected for simple diffuse injury and focal injury to help test our initial hypothesis. It is likely that a more general TBI population would have combinations of these injuries. We also note that, other than post-injury interval and age of survivors, we have no information about the nature of the injuries in the chronic TBI group. Finally, particularly for human studies, we need to point out that the interpretation of the functional relevance of circulating miRNA changes is based on their predicted effects on gene targets identified by miRNA target prediction algorithms. This informs and provides a cautionary note to our understanding of the potential functional roles played by any differentially expressed miRNAs in rat and human TBI.

The heterogeneity of the TBI population actually points to a reason why PCA of miRNA profiles may help identify TBI better than individual biomarkers. It is instructive to recognize that complex traits like intelligence, height and life expectancy are polygenic traits, i.e., there is no single gene that determines these characteristics; rather, the phenotype is due to huge numbers of common gene variants with tiny effect sizes^42^. In our study, PCA of transcriptome-wide miRNA expression profiles, including those with small but significant effect sizes, facilitated the clear identification of TBIs and TBI subgroups despite the variability among the patients. This type of analysis may prove to be valuable for identifying mild TBIs that are often missed at initial assessments but have been found to result in chronic cognitive impairment^43^. Our study also indicates that TBI leaves a long-lasting “miRNA fingerprint” in circulating biofluids which may serve to identify injured individuals across all post-injury intervals as well as portend risk of later neurodegenerative disorders.

To improve care for TBI, it is necessary to address the need for better characterization and mechanistic understanding of the life-long consequences of TBI, especially the cognitive and psychiatric sequelae^44^. Single outcome measures have been deemed insufficient to assess individuals after a TBI, thus multiple assessments are called for^1^. Since the idea that predictive analytics (i.e. predictive modeling, machine learning, data mining of genomic data) can improve medical care is increasingly acknowledged^45^, miRNA-sequencing of biofluids, which is becoming more cost-effective to perform, would be an ideal adjunct to current imaging modalities and neuropsychological tests^46^ that may aid in a precision-health approach towards better stratification of patients into subgroups as well as other brain disorders^8^.

## Supplementary information

Supplementary Materials.
