# Supplementary material for: MicroRNA sequencing of rat hippocampus and human biofluids identifies acute, chronic, focal and diffuse traumatic brain injuries
Source: Sci Rep. 2020 Feb 24;10:3341. doi: 10.1038/s41598-020-60133-z (PMC7040013; doi:10.1038/s41598-020-60133-z)
Supplement: Supplementary file 1 — Supplementary Materials. [file 41598_2020_60133_MOESM1_ESM.pdf]

Supplementary Materials

MicroRNA Sequencing of Rat Hippocampus and Human Biofluids Identifies Acute, Chronic, Focal and Diffuse Traumatic Brain Injuries

Harris A. Weisz[1], Deborah Kennedy[1], Steven Widen[1], Heidi Spratt[1], Stacy Sell[1], Christine Bailey[1], Melinda Sheffield-Moore[1], Douglas S. DeWitt[1], Donald S. Prough[1], Harvey Levin[2], Claudia Robertson[2], Helen L. Hellmich[1]

[1] The University of Texas Medical Branch at Galveston, Galveston, TX, USA

[2] Baylor College of Medicine, Houston, TX, USA

This PDF includes:

Supplementary Methods
Supplementary Table S1
Supplementary Figures S1 to S9
Supplementary Discussion
Supplementary References

Supplementary Methods

Animals
All animal experiments were approved by the Institutional Animal Care and Use Committee of the University of Texas Medical Branch, Galveston, Texas and conducted according to the National Institutes of Health Guide for the Care and Use of Laboratory Animals (8th edition, National Research Council).

For microRNA-sequencing experiments. adult male Sprague-Dawley rats (250 g–300 g) from vendor Charles Rivers (Portland, Maine) were housed 2 per cage with food and water ad libitum in a vivarium with these constant conditions: light cycle (6∶00–18∶00) temperature (21°C–23°C), and humidity (40%–50%). For each post-TBI interval, hippocampal microRNA expression was analyzed in four sham-injured and four TBI rats; thus, 56 rats were used for microRNA sequencing and digital droplet PCR analysis. For PCR array analysis of FPI and controlled cortical impact (CCI) adult male Wistar rats (250 g – 300 g) from Charles Rivers (Portland, Maine) were used and housed as described above.

Surgical Preparation
Rats were anesthetized with isoflurane in an anesthetic chamber, intubated, and mechanically ventilated with 1.5-2.0% isoflurane in O2: room air (70:30) using a volume ventilator (EDCO Scientific, Chapel Hill, NC). Rats were prepared for parasagittal fluid-percussion injury (FPI) as previously described [17,18]. Surgical preparation of sham-injured and TBI rats was identical except for the injury. Rats were placed in a stereotaxic head frame and the scalp was sagittally incised. A 4.0 mm diameter hole was trephined into the skull 2.0 mm to the right of the sagittal suture and midway between lambda and bregma. A modified Luerlok syringe hub was placed over the exposed dura, bonded in place with cyanoacrylic adhesive and covered with dental acrylic. Isoflurane was discontinued; rats were connected to the fluid-percussion trauma device and immediately after the return of a withdrawal reflex to paw pinch a severe (2.3 atm) FPI; was administered. After FPI or sham injury, rats were disconnected from the fluid-percussion device and the righting reflex (the time to right), was measured. Rats were then placed back on isoflurane (2%), wound sites were infused with bupivicaine and sutured with prolene. Isolflurane was discontinued and the rats were extubated and allowed to recover in a warm, humidified incubator.

Controlled cortical impact (CCI) was performed at Baylor College of Medicine as previously described in Cherian et al., 1996[19].

Microdissection of hippocampus
Tissue dissections were performed using sterile, RNase treated Iris tissue forceps. The two cerebral hemispheres of the cortex were opened along the cerebral longitudinal fissure and separated with the forceps to expose the hippocampus. Next, each hippocampus was individually separated from the cortex and midbrain using the same Iris tissue forceps and each placed into separate sterile Eppendorf tubes containing RNAlater (Thermo Fisher Scientific) and stored at 4°C or placed into separate empty sterile Eppendorf tubes and fresh frozen on dry ice for 3-5 minutes.

Rodent serum isolation and PCR array analysis

Serum from rats subjected to FPI or CCI (n=3/control or TBI group, n=12 total) was used for miRNA isolation using miRNeasy Serum/Plasma Kit (Qiagen). To start, 200 µl of serum was removed from freezer storage (-80°C) and thawed. Five volumes of QIAzol Lysis Reagent (1000 µl) was added to the sample and mixed thoroughly by pipetting up and down. Samples were incubated for 5 minutes at room temperature (15–25°C), then 3.5 µl of the miRNeasy Serum/Plasma Spike-In Control (1.6 x 10^8 copies/µl working solution) was added and mixed thoroughly. Two-hundred µl of chloroform was added to the sample and it was vortexed vigorously for 15 seconds. Once mixed, the samples were centrifuged for 15 minutes at 12,000 x *g* at 4°C. Carefully, the upper aqueous phase was moved to a new while avoiding disrupting the interphase and 1.5volumes of 100% ethanol were added and mixed thoroughly by pipetting up and down. The samples were then pippeted into an RNeasy MinElute spin column, 700 µl at a time, and centrifuge at 10,000 x g for 15 seconds at room temperature (15–25°C) discarding the flow through after, until all of the sample had been passed through. From there, 700 µl of Buffer RWT was added to the spin column and centrifuged for 15 seconds at 10,000 x g, and the flow through was discarded. This was followed by a wash with 500 µl of Buffer RPE and centrifugation of 15 seconds at 10,000 x *g*. The flow through was discarded and 500 µl of 80% ethanol was added to the spin column followed by a 2-minute centrifugation at 10,000 x g. The spin column was then placed into a new collection tube and spun for 5 minutes at maximum speed (13,000 x g). Afterwards, the column was placed into another new collection tube and 14 µl of nuclease free water was added directly to the center of the filter and was then centrifuged at maximum speed for 1 minute to elute the RNA.

PCR array analysis was performed using miScript miFinder PCR arrays (Qiagen) per manufacuter's protocol. Initially, 5 µl of total RNA was reverse transcribed using the miScript II RT Kit (Qiagen) and then pre-amplification of the RT product was performed using the miScript PreAMP PCR Kit and Primer Mixes (Qiagen), per manufacturer's protocol. PCR was performed using the miScript SYBR Green PCR Kit and miScript miFinder Arrays (MIRN-001Z; Qiagen) on a Roche LightCycler 96 (Roche Life Science).

Acquisition of Patient Samples

Blood samples were collected from TBI patients 24 (n = 21), 48 (n = 7), and 96 hours (n = 5) post-injury. Samples were collected in vacutainer SST tubes. Blood samples were allowed to clot, spun down, and the serum was placed in 1 ml aliquots in RNase-free tubes. Serum samples from chronic TBI patients (n = 6) were obtained through a collaboration with Dr, Melinda Sheffield-Moore at the University of Texas Medical Branch, Galveston, TX. Healthy control serum samples (n = 6) were obtained prospectively from BioreclamationIVT (http://www.bioreclamationivt.com/). Serum samples were frozen and stored at -80°C until used for RNA isolation and genomic analyses.

Human serum miRNA isolation and precipitation

Approximately 1ml of human serum was used for miRNA isolation using the miRVana Paris Kit (#AM1556; Invitrogen) following manufactures protocol for extraction from biofluids. RNA extractions were performed from 500 µl of patient serum in tandem (total 1 ml of patient sample used for each isolation). Five-hundred µl of lysis solution solution was added to each 500 µl aliquot of sample and incubated on ice for 5 minutes. Phenol-Chloroform was added at 2X the

volume of sample plus lysis solution (1 ml) to equal 2 ml total volume. The samples were vortexed vigourously for approximately 30 seconds before being centrifuged at 10,000 xg for 5 minutes. After centrifugation the aqueous phase was removed, and the volume recorded. One-third volume of 100% ethanol was added to each sample, mixed thoroughly, and then added to the spin column in 700 µl aliquots until all of the sample was passed through the column. Samples were spun for 30 seconds each time at 10,000 xg and the volume was recorded. Two-thirds volume of 100% ethanol was then added to the samples. Again, samples were mixed thoroughly, and then added to the spin column in 700 µl aliquots until all of the sample was passed through the column (30 seconds each time at 10,000 xg). On the last spin the flow through was discarded and 700 µl of Wash Solution #1 (provided with kit) was passed through the column (30 seconds each time at 10,000 xg). Afterwards, the flow through was discarded and then 500 µl of Wash Solution #2 (provided with kit) was passed through the column (30 seconds each time at 10,000 xg). The flow through was discarded and the column spun for an additional minute to remove any residual fluid. The column was then placed in a sterile, RNase-free tube and 100 µl of 95°C nuclease free water was placed directly on the filter to elute the miRNA (30 seconds each time at 10,000 xg).

The 2, 100 µl aliquots of the same patient samples were then added together to ethanol precipitate the miRNA to make a highly concentrated solution. To the now 200 µl solution, I added 0.01 volumes of 3M sodium acetate and 2.5 volumes of ice-cold 100% ethanol. The sample was vortexed thoroughly and precipitated in a combination bath of dry ice and ethanol until completely frozen (approximately 1 hour). The frozen solution was then centrifuged at 4°C for 10 minutes at 13,000 xg. The supernatant was carefully discarded as to not disturb the pellet which had formed. The pellet was then washed twice with 0.5 ml of ice cold 75% ethanol, after each wash it was spun at 4°C for 10 minutes at 13,000 xg. After the final spin the ethanol was removed and the pellet allowed to air dry. Once all liquid had evaporated or was removed and the pellet dried, 10 µl of nuclease free water was added and the pellet resuspended.

miRNA sequencing
Samples were sent to the UTMB Sequencing Core for processing. First, miRNA samples were quantified using a Qubit fluorescent assay. Libraries for sequencing were prepared with the NEBNext Small RNA Library Prep (New England BioLabs, Inc.) following the manufacturer's protocol. Briefly, linker molecules are ligated to each end of the small RNAs then reverse transcriptase is used to make a cDNA copy. Adapter are added and the library is amplified by PCR. Index sequences are included to identify individual samples. Polyacrylamide-gel purification was used to enrich for 22 nt miRNA sequences.

Sequencing was performed on a NextSeq 550 (Illumina, Inc.) with the High-Output flowcell using a single-end 75 base sequencing protocol with the SBS v2 sequencing kit. Fastq files were processed using the miRDeep2 program, version 2.0.0.8. Adapter sequences were trimmed off and duplicate reads were collapsed into fasta format files using the miRDeep2 mapper.pl function with default parameters. Reads shorter than 18 bases were excluded. The quantifier.pl function was used to map the reads to mature and precursor miRNA sequences downloaded from the online miRBase database, version 21. The –W parameter was used to distribute multi-mapping read counts between miRNAs, then the counts for miRNAs with the same mature sequence were summed.

microRNA target prediction and bioinformatics analysis
To infer the regulatory actions of miRNA from the sequencing data and infer whether these miRNAs are representative of brain pathology we performed an iterative process for miRNA target prediction. Initially, miRNAs that were significant across all four statistical tests in R in separate time intervals were loaded into a Venn diagram analysis suite (Venny 2.1; Oliveros JC 2015)[20] to find the overlapping significant miRNAs. The overlapping miRNAs were then separated into top 10 miRNAs (either up or downregulated) based on the adjusted *p* value. The miRNAs in these lists were then input into a miRNA target prediction online suite, miRDB [21]. miRDB uses miRBase V22[22] as its source miRNA sequence database for target prediction. Based on sequence data miRDB then uses a proprietary bioinformatics program, miRTarget, to assess functional annotations of these miRNA and gene targets. miRTarget uses constructive machine learning procedures known as support vector machines and high-throughput training datasets to generate a target prediction score between 50-100. The higher the target prediction score the more confidence there is in the predicted relationship between miRNA and target. Genes were filtered to show only those with a target score > 60. After filtering, the lists of genes were uploaded into IPA (Qiagen) and an ‘Expression Analysis’ was performed. For the Expression Analysis, genes were filtered by confidence level (Experimentally Observed and High Predicted) and by species (Human, Rat, and Mouse) and included evidence from all tissue and cell types. Pathways are determined by the overlap of genes from our uploaded dataset and the reference set in IPA (Fishers Exact Test). A subsequent analysis between focal and diffuse injury with data from the 24 h cohort was performed in the same manner. The top canonical pathways were selected based on relevance to central nervous signaling or involvement in immune system related processes (e.g., inflammation). A comparison analysis of signaling pathways across all human TBI time-intervals, focal and diffuse injury subtypes, and CCI and FPI data sets was performed to identify similarities that may exist in pathways of predicted target genes. The color intensity corresponds to the level of significance and is calculated as the -log of the Fisher’s Exact p value. The darker the color the more highly significant that pathway is for that individual analysis. Pathways relevant to nervous system signaling were selected from a larger list that included all predicted potential pathways.

Droplet Digital PCR
Digital Droplet PCR (ddPCR; Bio Rad Inc.) was performed for absolute quantification of miRNA concentrations in rat serum after FPI. ddPCR provides stand-alone absolute quantification of low-abundance miRNA with improved sensitivity and precision compared to conventional qPCR. For reverse transcription and pre-amplification, the Taqman Advanced miRNA cDNA Synthesis Kit (Thermofisher Scientific Inc.) was used. Per the recommended protocol, the reverse transcription (RT) of serum derived miRNA with the maximum allowed volume of total RNA per reaction. Beginning with poly(A) tail addition, 2 µl of total RNA was added to a reaction tube with other poly(A) reaction components. A thermal cycler was set to incubate at 37°C for 45 minutes; 65°C for 10 minutes; and then held at 4°C until the next step. After addition of the poly(A) tail an adaptor ligation reaction was performed by adding the 5 µl poly(A) reaction to 10 µl of the master mix of the adaptor ligation reaction. The entire reaction was placed in the thermal cycler and set to 16°C for 60 minutes; then held at 4°C. For the RT reaction, the 15 µl adaptor ligation reaction was added to 15 µl of RT reaction master mix and placed into a thermal cycler and incubated at 42°C for 15 minutes; 85oC for 5 minutes; and held

at 4ºC. For the miR-Amp reaction 5 µl of the RT reaction product was added to 45 µl of miR-Amp reaction mix and incubated in a thermal cycler at 95ºC for 5 minutes; followed by 14 cycles at 95ºC for 3 seconds and 60ºC for 30 seconds; 1 cycle at 99ºC for 10 minutes; then held at 4ºC. For the ddPCR reaction 1.5 µl of the miR-Amp reaction, including a non-template control, was added to 20.5 µl of ddPCR reaction mix which contains the miRNA assay of choice, ddPCR supermix, and RNase free water. Droplets were then generated by adding 20 µl of the final miR-Amp reaction with 70 µl of droplet oil in a droplet generator (Bio Rad Inc.). The droplet generator produces approximately 20,000 droplets per sample, each containing an individual miRNA transcript and PCR reaction reagents. The PCR reaction was performed in a C1000 (Eppendorf) thermal cycler. The final PCR product contained within the droplets were then read in a CFX1000 (Bio Rad Inc.) in duplicate. Droplets are measured as positive or negative based a on a fluorescence amplitude threshold. Concentrations of the target and reference miRNAs are determined by the number of positive and negative droplets and their Poisson-based 95% confidence interval. Only wells that contain at least 10,000 droplets are used in concentration calculations. Data from the droplet reader is then analyzed using Quantasoft software where thresholds for fluorescence can either be set automatically or manually by the user. Concentrations of miRNA are reported as copies/µl.

Data Analysis
For miScript PCR array analysis the expression of each miRNA was modeled by analysis of variance; miRNA expressions as $2^{(\text{Avg. } (\text{Delta(Ct)})}$ were log2 transformed to an approximation of the normal distribution prior to modeling. Differences among treatment groups (Naive, CCI, FPI) were assessed by Tukey-adjusted contrasts, followed by Benjamini-Hochberg control of the false discovery rate (FDR) among the miRNAs at the 5% level.

Raw data produced by miRNA-sequencing of sham and TBI hippocampus were input into DESeq2 or EdgeR and normalized to the reads per kilo base per million mapped (RPKM). This provided a 'scaling factor' which was applied to all time-specific samples. Differential expression analysis of the miRNA-seq counts was done using two R packages: Empirical Analysis of Digital Gene Expression Data in R (edgeR) and Differential gene expression analysis based on the negative binomial distribution (DESeq). Both use the negative binomial distribution in order to assess differences in miRNA expression using count data and a general linear model (glm) analysis framework. For the edgeR package, counts and a grouping string were read in. The miRNA that had low expression across all groups were filtered using a count per million of 1. The glm procedure was used to estimate two exact tests: the quasi-likelihood (QL) F-test (the Wald Test) and the likelihood ratio test (LR test). The top tags from each were read out and compared. For the DESeq2 package, counts and their grouping string were read in. miRNA were filtered using a count of less than eight across each miRNA. A quasi-likelihood (QL) F-test (the Wald Test) and the likelihood ratio test (LR test) were run. The top tags were read for each and compared.

For DDPCR experiments a Wilcoxon Rank Sum test was performed to assess statistical significance between TBI and sham injury groups.

Table S1. Immunoregulatory roles of TBI-dysregulated rat miRNAs

miR-155-5p	Roles in T cell functionality[23]<br>Inflammatory signaling[24]<br>Redox signaling[25]
miR-223-5p	PPARγ signaling[26]<br>Inflammasome signaling[27]
miR-21-5p	BBB stability[28]<br>Anti-apoptosis[29]<br>Inflammatory signaling[30]
miR-146a-5p	NFκB signaling[31]
miR-142-5p	Regulated by cytokine expression[32]

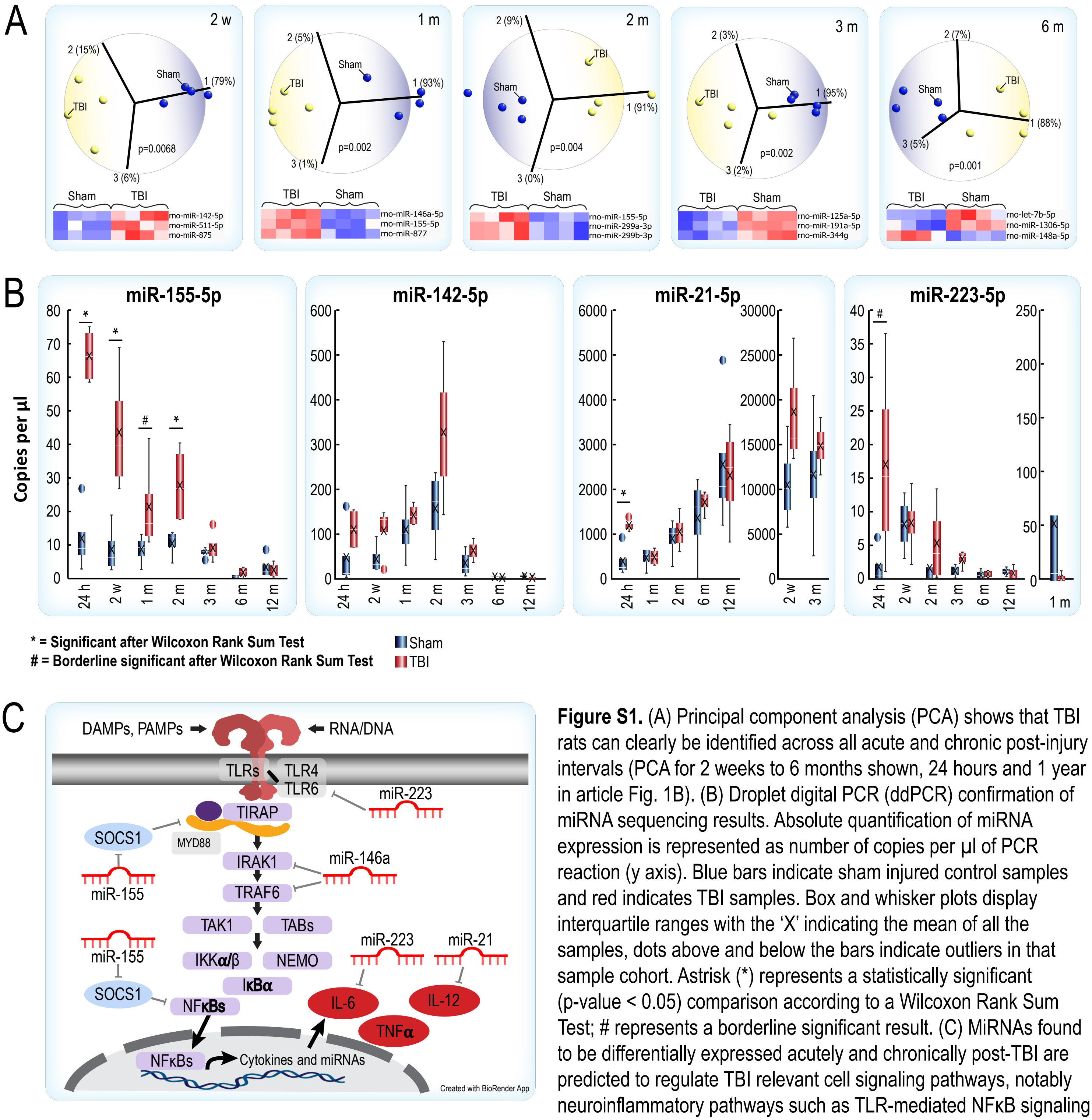

Figure S1. (A) Principal component analysis (PCA) shows that TBI rats can clearly be identified across all acute and chronic post-injury intervals (PCA for 2 weeks to 6 months shown, 24 hours and 1 year in article Fig. 1B). (B) Droplet digital PCR (ddPCR) confirmation of miRNA sequencing results. Absolute quantification of miRNA expression is represented as number of copies per µl of PCR reaction (y axis). Blue bars indicate sham injured control samples and red indicates TBI samples. Box and whisker plots display interquartile ranges with the 'X' indicating the mean of all the samples, dots above and below the bars indicate outliers in that sample cohort. Astrisk (*) represents a statistically significant (p-value < 0.05) comparison according to a Wilcoxon Rank Sum Test; # represents a borderline significant result. (C) MiRNAs found to be differentially expressed acutely and chronically post-TBI are predicted to regulate TBI relevant cell signaling pathways, notably neuroinflammatory pathways such as TLR-mediated NFκB signaling.

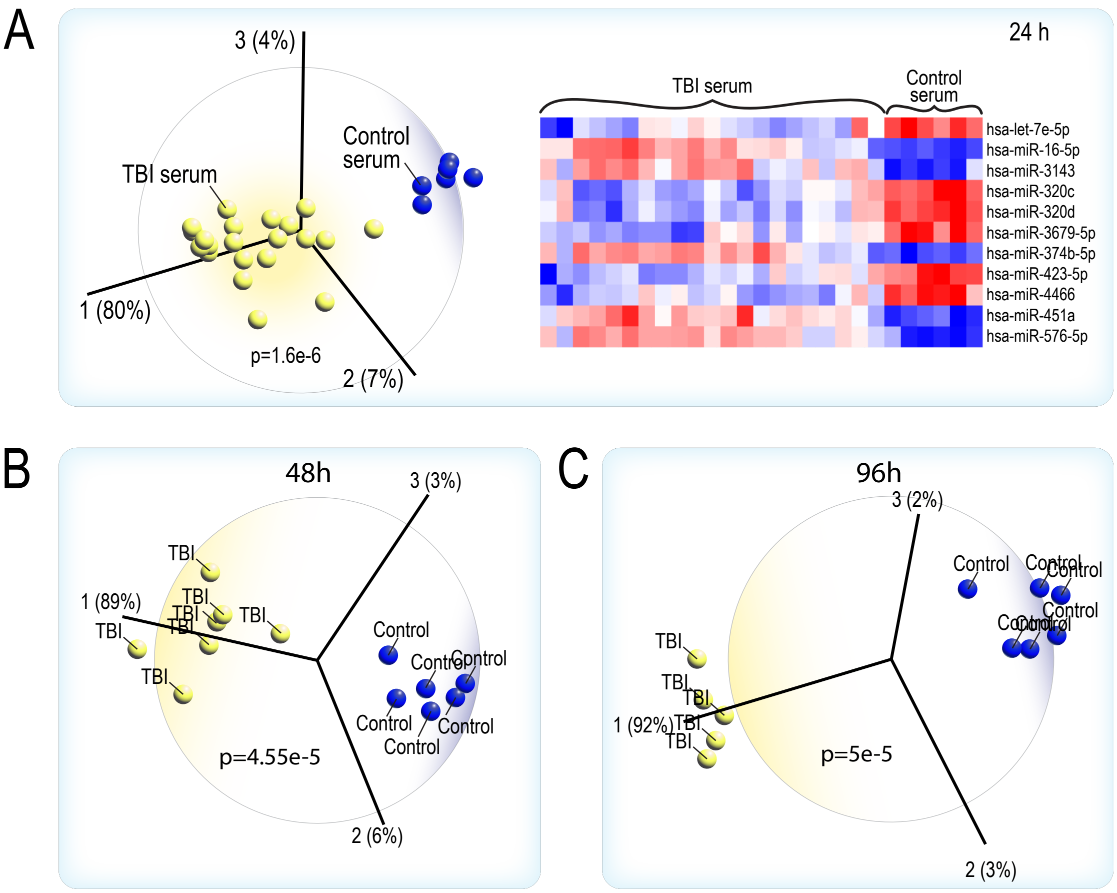

Figure S2. (A) Principal component analysis (PCA) and the hierarchical clustering heatmap reveals serum miRNAs that discriminate between TBI and healthy, age-matched controls in the 24-hour cohort. The first three principal components represent 91% of the variance in the miRNA-seq data. PCA clearly distinguishes the (B) 48-hour TBI cohort from uninjured controls and the (C) 96-hour TBI group from uninjured controls. The first three principal components represent 98% (48 hours) and 97% (96 hours) of the variance in the miRNA-seq data.

A

48 hours

Up		
miRNA	log2 Ratio	FDR
hsa-miR-1260b	3.88	1.23e-05
hsa-miR-7704	5.87	1.08e-04
hsa-miR-499a-5p	2.47	5.33e-04
hsa-miR-451a	2.39	5.33e-04
hsa-miR-4433b-5p	3.01	8.34e-04
hsa-miR-1260a	2.54	9.07e-04
hsa-miR-4488	4.51	1.21e-03
hsa-miR-129-5p	3.05	1.36e-03
hsa-miR-185-5p	1.58	1.69e-03
hsa-miR-335-5p	3.28	2.13e-03

Down		
miRNA	log2 Ratio	FDR
hsa-let-7e-5p	-1.83	2.51e-03
hsa-miR-3679-5p	-2.58	1.08e-04
hsa-miR-125a-5p	-2.78	1.16e-06
hsa-miR-204-5p	-2.82	8.17e-03
hsa-miR-1228-5p	-3.73	1.16e-06
hsa-miR-6891-5p	-4.23	4.42e-03
hsa-miR-4466	-4.77	1.81e-07
hsa-miR-135a-5p	-5.36	9.97e-03
hsa-miR-6740-5p	-6.04	6.85e-04

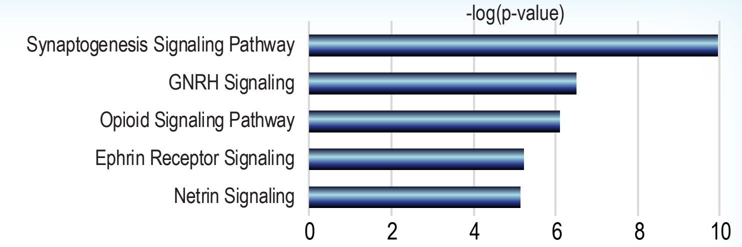

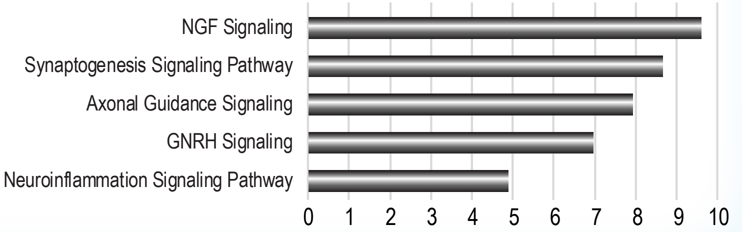

B

96 hours

Up		
miRNA	log2 Ratio	FDR
hsa-miR-1260b	4.58	7.34e-10
hsa-miR-185-5p	2.26	3.85e-07
hsa-miR-16-5p	2.45	2.50e-06
hsa-miR-7704	6.04	3.52e-06
hsa-miR-7641	2.40	3.52e-06
hsa-miR-186-5p	2.21	3.52e-06
hsa-miR-4433b-5p	3.67	6.90e-06
hsa-miR-30d-5p	1.34	7.61e-06
hsa-miR-425-5p	1.71	9.14e-06
hsa-miR-199b-5p	3.83	9.73e-06

Down		
miRNA	log2 Ratio	FDR
hsa-miR-215-5p	-3.16	1.21e-11
hsa-miR-1228-5p	-3.82	5.67e-08
hsa-let-7e-5p	-2.67	3.00e-07
hsa-miR-4466	-4.10	2.50e-06
hsa-miR-125a-5p	-2.83	3.52e-06
hsa-miR-320c	-2.05	9.73e-06
hsa-miR-320d	-2.29	3.41e-05
hsa-miR-10b-5p	-1.95	1.86e-04
hsa-miR-483-5p	-2.88	2.26e-04
hsa-miR-10a-5p	-1.86	4.62e-04

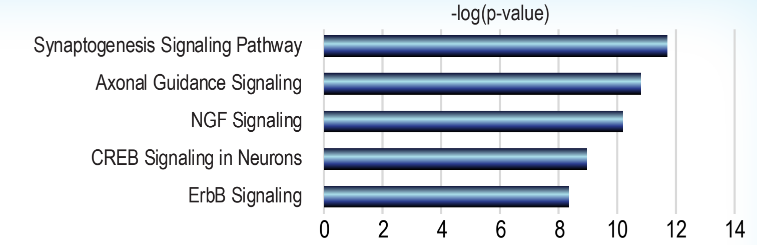

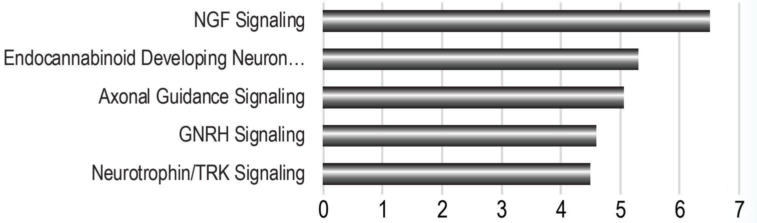

Figure S3. The top 10 up- and down-regulated miRNAs by level of significance (FDR adjusted p-value) for the 48-hour (A) and 96-hour (B) patient cohorts. Ingenuity Pathway Analysis of predicted miRNA gene targets show the top nervous system-related canonical pathways for the up and down miRNAs, respectively. The x-axes in (A) and (B) are based on the -log of the Fisher’s Exact p-value.

A

Focal

Up

miRNA	log2 Ratio	FDR
hsa-miR-30d-5p	1.18	2.61e-07
hsa-miR-191-5p	1.66	3.15e-06
hsa-let-7g-5p	1.70	1.39e-05
hsa-miR-503-5p	1.70	1.52e-05
hsa-miR-660-5p	1.01	1.38e-04
hsa-miR-15b-5p	2.67	1.47e-04
hsa-miR-17-5p	1.52	1.47e-04
hsa-miR-425-5p	1.33	1.47e-04
hsa-miR-18a-5p	2.73	2.75e-04
hsa-miR-454-5p	2.28	4.16e-04

Down

miRNA	log2 Ratio	FDR
hsa-miR-320b	-1.75	3.91e-06
hsa-miR-6765-5p	-4.41	6.63e-06
hsa-miR-516b-5p	-2.51	8.50e-06
hsa-miR-4429	-2.07	1.34e-05
hsa-miR-204-5p	-2.18	1.52e-05
hsa-miR-99b-5p	-1.33	1.25e-04
hsa-MIR-139-5p	-1.31	1.75e-04
hsa-miR-342-5p	-1.21	4.36e-04
hsa-miR-6862-5p	-2.74	1.07e-03
hsa-miR-526b-5p	-4.61	2.78e-03

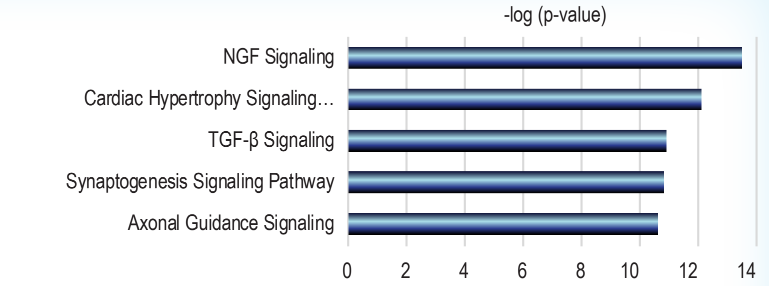

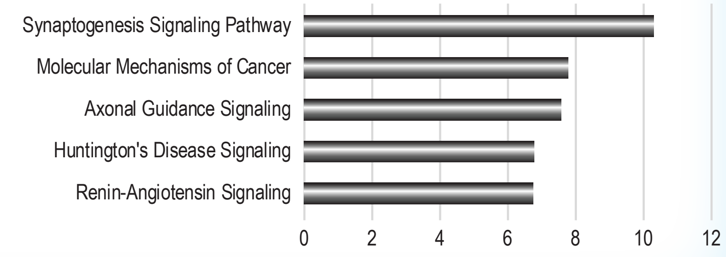

B

Diffuse

Up

miRNA	log2 Ratio	FDR
hsa-miR-509-3-5p	5.38	4.77e-05
hsa-miR-100-5p	1.23	2.84e-03
hsa-miR-195-5p	2.48	3.99e-03
hsa-miR-378a-5p	2.21	7.93e-03
hsa-miR-216b-5p	3.79	1.38e-02
hsa-miR-4792	2.76	1.57e-02
hsa-miR-4662a-5p	3.45	1.66e-02
hsa-miR-574-5p	4.30	2.04e-02
hsa-miR-218-5p	2.35	2.16e-02
hsa-miR-223-5p	1.80	2.32e-02
hsa-miR-324-5p	3.02	2.83e-02

Down

miRNA	log2 Ratio	FDR
hsa-miR-412-5p	-2.45	4.46e-03
hsa-miR-9-5p	-1.73	2.73e-03

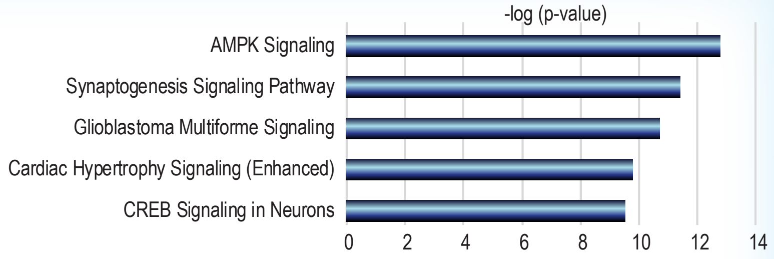

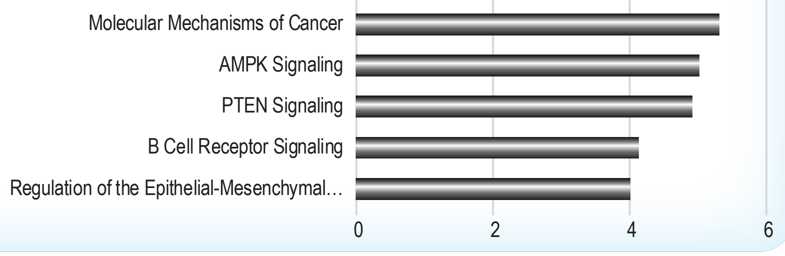

Figure S4. miRNA expression in focal (A) and diffuse (B) injury patients in the 24-hour, post-injury cohort were compared to healthy controls by LRT and Wald tests in EdgeR and DESeq2. The top 10 up- and down-regulated overlapping significant miRNAs (by level of significance from the LRT and Wald tests) are listed. Next to each list are the top canonical pathways (Fisher's Exact Test; p-value < 0.05) represented by genes predicted to be regulated by these miRNAs.

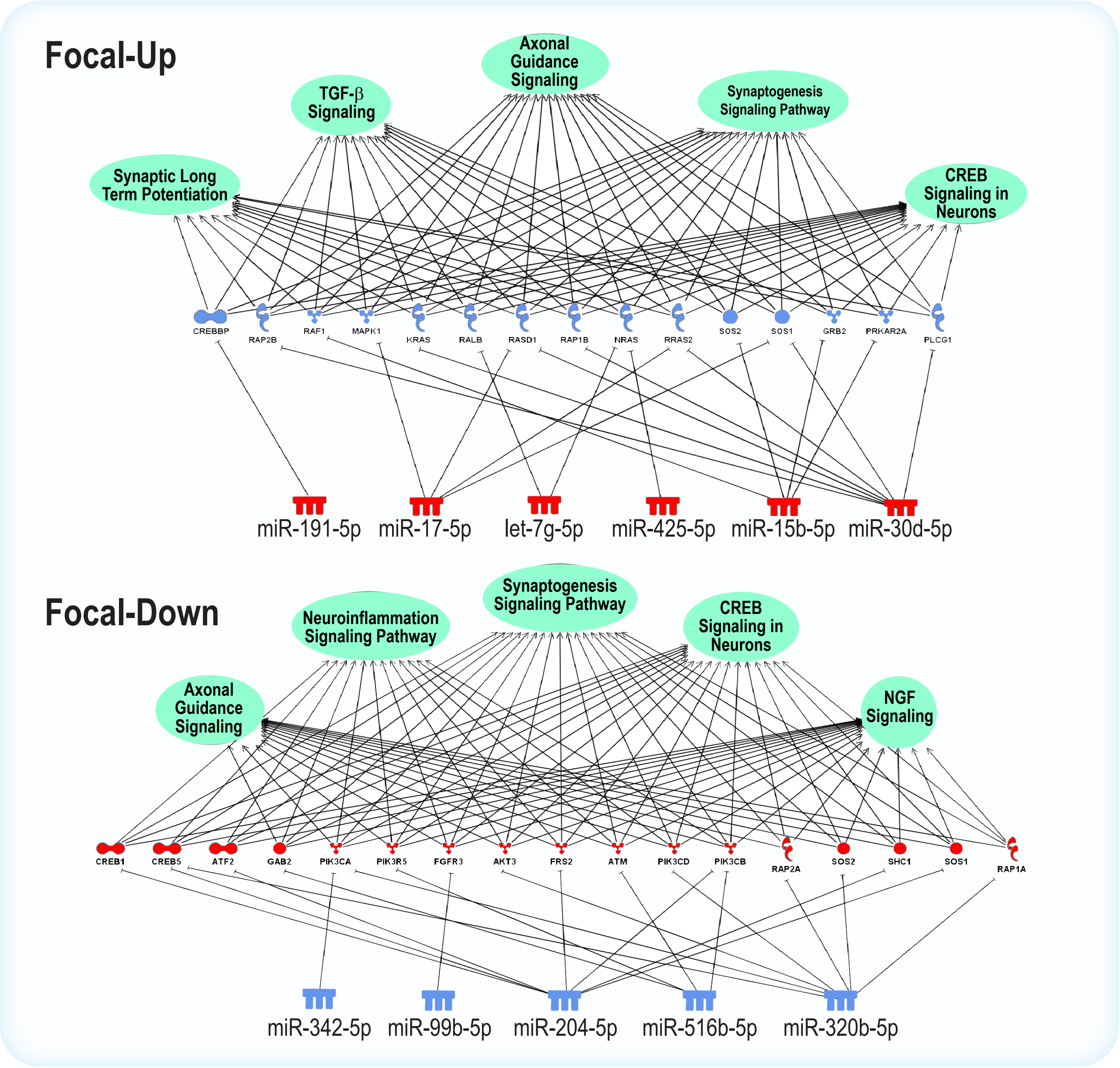

Figure S5. Ingenuity Pathway analysis reveals predicted gene targets enriched in predominately nervous system signaling pathways for the 24-hour focal injury cohort. miRNA colors signify increased (red) or decreased (blue) expression compared to control samples. The color of the gene targets represents the predicted direction of regulation based on target-prediction using miRDB. The green circles represent biological pathways comprising of predicted genes based on Ingenuity Pathway Analysis. Data were analyzed through the use of IPA (QIAGEN Inc., https://www.qiagenbioinformatics.com/products/ingenuitypathway-analysis).

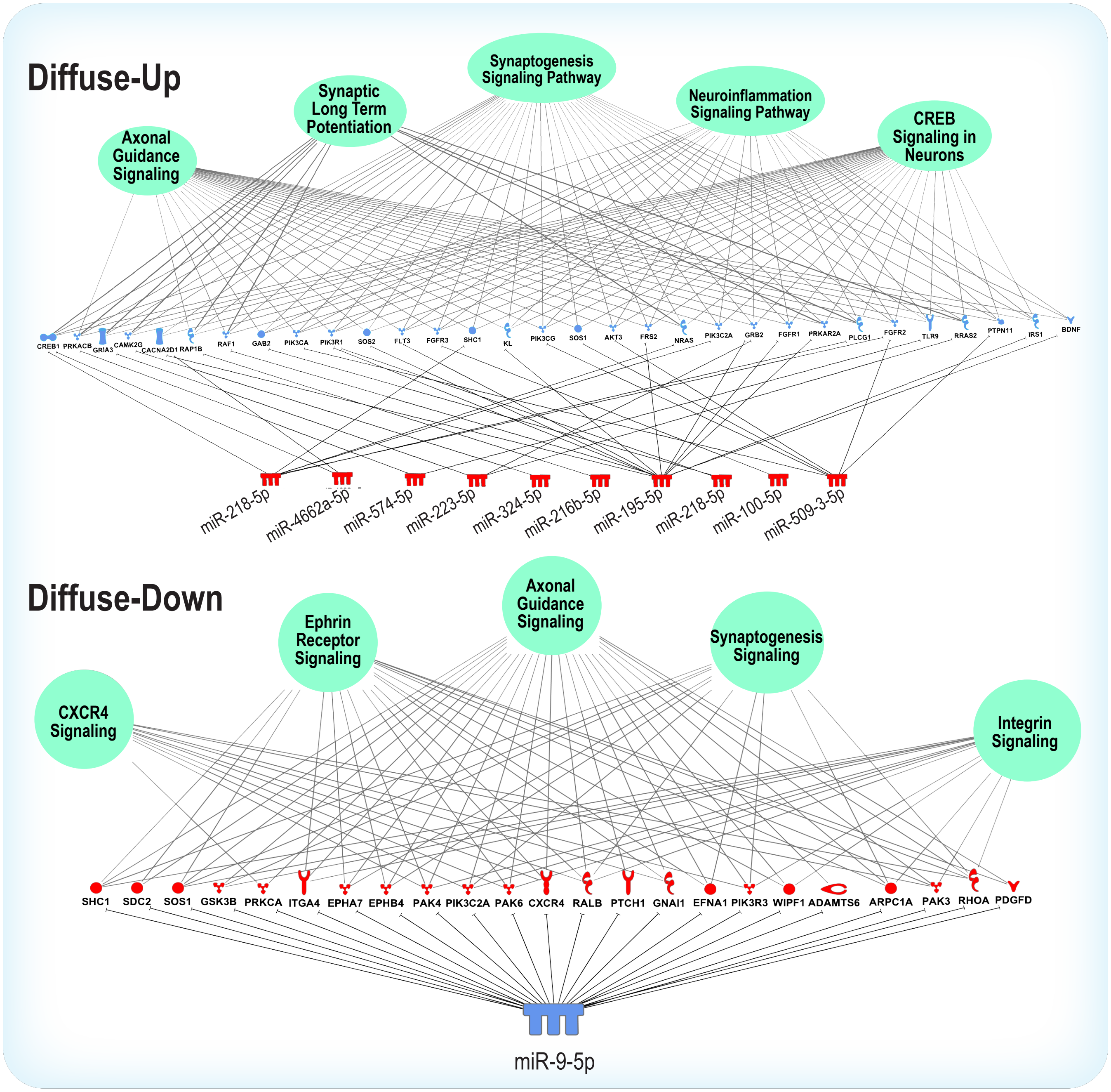

Figure S6. Ingenuity Pathway analysis reveals predicted gene targets enriched in predominately nervous system signaling pathways for the 24-hour diffuse injury cohort. miRNA colors signify increased (red) or decreased (blue) expression compared to control samples. The color of the gene targets represents the predicted direction of regulation based on target-prediction using miRDB. The green circles represent biological pathways comprising of predicted genes based on Ingenuity Pathway Analysis. Data were analyzed through the use of IPA (QIAGEN Inc., https://www.qiagenbioinformatics.com/products/ingenuitypathway-analysis).

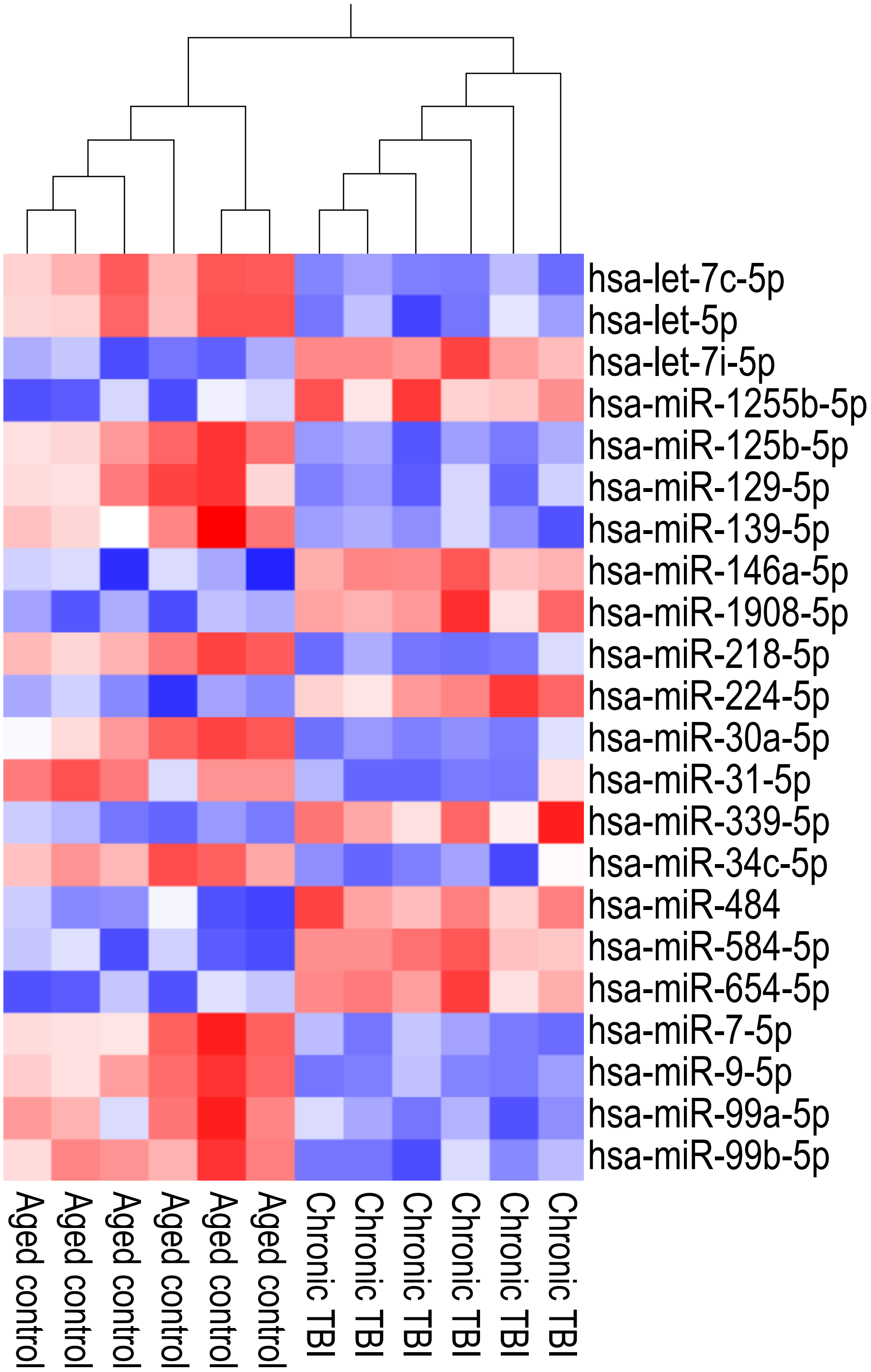

Figure S7. Qlucore Omics Explorer was used to generate a hierarchical clustering heatmap of serum microRNA expression in chronic TBI and age-matched controls. p=7.8e-4, q=0.05.

Up		
**miRNA**	**log2 Ratio**	**FDR**
hsa-miR-451a	3.56	1.59e-04
hsa-miR-1908-5p	6.91	2.72e-04
hsa-miR-224-5p	5.57	7.36e-04
hsa-let-7i-5p	2.94	7.36e-04
hsa-miR-654-5p	9.12	8.03e-04
hsa-miR-146a-5p	3.66	8.03e-04
hsa-miR-584-5p	4.27	1.07e-03
hsa-miR-1255b-5p	8.77	1.74e-03
hsa-miR-25-5p	8.65	2.14e-03
hsa-miR-423-5p	2.78	3.52e-03

Down		
**miRNA**	**log2 Ratio**	**FDR**
hsa-miR-9-5p	-7.30	1.75e-05
hsa-miR-218-5p	-7.48	7.72e-05
hsa-miR-346	-8.42	9.64e-05
hsa-miR-708-5p	-9.55	9.64e-05
hsa-miR-383-5p	-7.40	1.07e-04
hsa-miR-31.5p	-7.47	1.80e-04
hsa-MIR-219a-5p	-8.06	2.72e-04
hsa-miR-34c-5p	-5.27	7.36e-04
hsa-miR-149-5p	-6.71	8.03e-04
hsa-miR-125b-5p	-3.56	1.07e-03

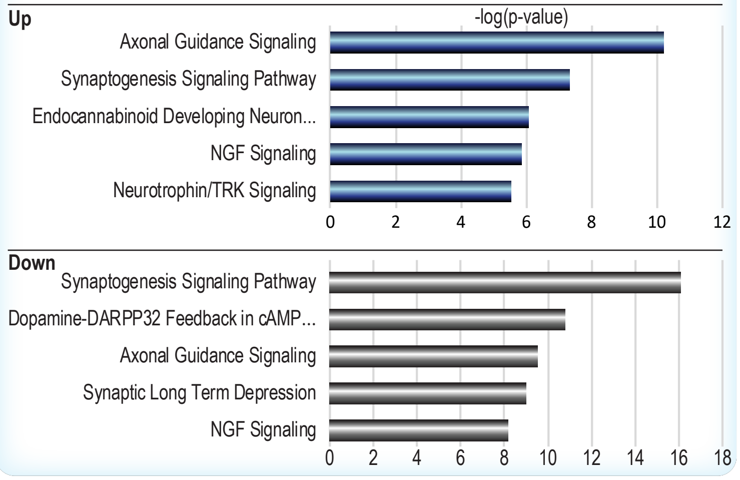

Figure S8. The top 10 up-regulated and down-regulated serum miRNAs by level of significance (FDR adjusted p-value) in the chronic TBI cohort and Ingenuity Pathway Analysis of the top nervous system-related canonical pathways for their predicted gene targets. The x-axes in the pathway graphs are based on the -log of the Fisher's Exact p-value.

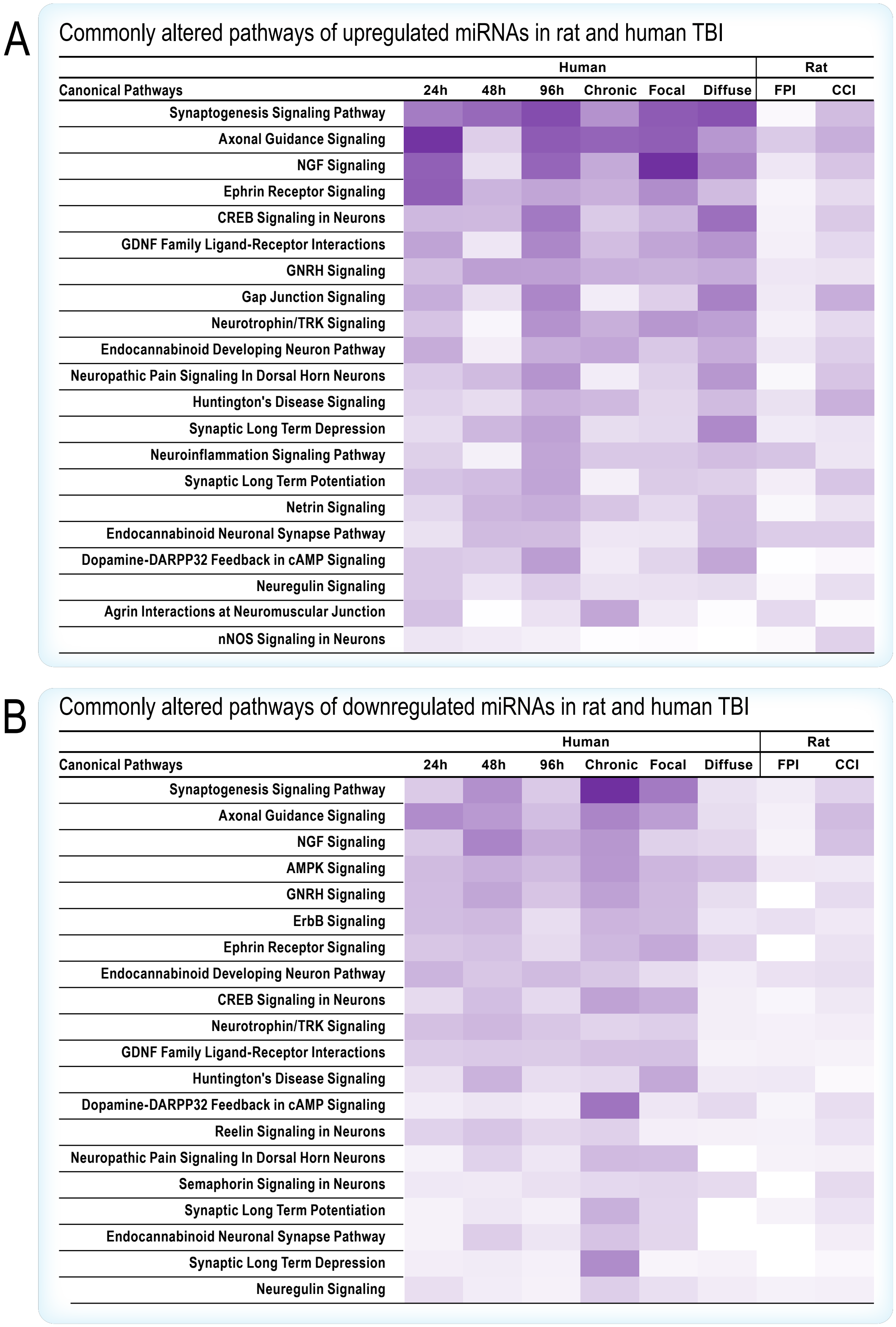

Figure S9. A comparison analysis of signaling pathways across all human TBI time-intervals, focal and diffuse injury subtypes, and rat CCI and FPI data sets was performed to identify similarities that may exist in pathways of predicted target genes. Pathways from gene targets of upregulated miRNAs in (A) and from downregulated miRNAs in (B) The color intensity corresponds to the level of significance and is calculated as the -log of the Fisher's Exact p value (used to determine whether the overlap of genes in the user data set and reference data set is greater than just by chance). The darker the color the more highly significant that pathway is for that individual analysis. Pathways relevant to nervous system signaling were selected from a larger list that included all predicted potential pathways.

Supplemental Discussion

Altered expression of miRNAs in TBI pathophysiology

Aberrant miRNA expression is a common phenomenon in many neurologic and neurodegenerative diseases. Within the last decade, studies examining miRNA expression after TBI have found altered expression profiles suggesting a potential role its pathophysiology. Two key studies published in 2009 by Lei et al. [33] and Redell et al., were the first to detail alterations of miRNA profiles in rat cerebral cortex (post-FPI) and hippocampus (post-CCI), respectively. Each of their studies were performed at acute time intervals after injury, ranging from 3 hours to 72 hours post-TBI. Redell and colleagues used qRT-PCR to validate differential expression of several miRNAs including miR-107, miR-130a, miR-223, miR-292-5p, miR-433-3p, miR-451, miR-541, and miR-711 [34]. At the time, the authors performed network analysis using predicted targets of these validated hippocampal miRNAs to infer their regulatory roles in various biological processes (signal transduction, transcriptional regulation, proliferation, and differentiation) known to be initiated after TBI or believed to affected by TBI [34]. In the Lei et al study, moderate FPI induced similar changes in the peri-contusional region of the injured cortex, though no gene ontology (GO) analysis was performed on predicted targets. Hu et al., (2012) showed that miRNAs are differentially expressed up to 7 days post-CCI [35]. Through GO enrichment and clustering analysis, they postulated that miRNAs induced acutely after CCI (24 hours) in the hippocampus, cooperatively regulate genes involved with pathological changes and stress management, while post-acute (7 days after CCI) miRNAs were more involved with brain repair mechanisms. Later, studies using a similar miRNA microarray approach found that a mild CCI acutely altered miRNA expression in the contused cortex [36]. All of the studies support the hypothesis that miRNAs are altered acutely after TBI. Although prior studies have noted the importance of miRNAs that are altered in the hippocampus up to 3-months after fluid percussion injury [37], there are no studies of miRNA changes in chronic TBI. The purpose of this current rat and human study is to address a large gap in knowledge that still exists pertaining to the magnitude and timing of miRNA changes in chronic TBI.

Previous studies identified miRNAs that are useful for differentiating between injured and uninjured groups in different models of TBI and miRNA profiles have been explored for their utility in differentiating between injury severities [38] [39] [40] [41] [42]. To our knowledge, there are no existing studies describing the differential identification of TBI subtypes as described in our current report.

Studies examining miRNA expression in human biofluids are primarily in acute TBI. Our study supports reports of differential miRNA expression from previous observations. For example, Redell et al., [43] assayed human TBI plasma and described upregulation of miRNAs including miR-16 in mild TBI (but found decreased levels in severe TBI), which we found persistently and significantly upregulated in our study. More recently, Di Pietro et al (2017) [41] found miR-425 was elevated in early time points after mTBI. In agreement with this finding, our data demonstrate miR-425 to be persistently and significantly elevated across all acute time points. A pilot study by Mitra et al. [44] found elevated levels of miR-142 and miR-423 were associated with the development of post-concussive syndrome. Similarly, our data show miR-142 is significantly elevated at 24 hrs post-ED admission compared to healthy controls (log2 ratio: 2.22; FDR adjusted p-value < 0.0001). All these data must be interpreted with caution because different

types of controls were used in previous studies compared to ours. The notion that a single miRNA or combination of miRNAs could be used to determine patient outcomes after TBI is an exciting prospect for prognostic capabilities in the future. A study by Papa et al. [46] described a panel of miRNAs that were significantly elevated in athletes post-season compared to pre-season, but also in athletes compared to non-athletes. Their data showed that even players with no reported concussions showed increased levels of miRNAs compared to controls and this correlated with lower scores on neurocognitive assessments. Those findings are consistent with other work suggesting that neurocognitive changes might occur even in players without clinically diagnosed concussions, potentially due to repetitive, subconcussive hits [47 48]. Taken together, miRNA profiling may be useful as an adjunct measure with neurocognitive testing in patients without objective imaging to confirm a TBI.

Supplementary References

References for Figure 5C

1. Stylli SS, Adamides AA, Koldej RM, et al. miRNA expression profiling of cerebrospinal fluid in patients with aneurysmal subarachnoid hemorrhage. *J Neurosurg* 2017; **126**(4): 1131-9.
2. McKeever PM, Schneider R, Taghdiri F, et al. MicroRNA Expression Levels Are Altered in the Cerebrospinal Fluid of Patients with Young-Onset Alzheimer's Disease. *Mol Neurobiol* 2018; **55**(12): 8826-41.
3. Boese AS, Saba R, Campbell K, et al. MicroRNA abundance is altered in synaptoneurosomes during prion disease. *Mol Cell Neurosci* 2016; **71**: 13-24.
4. O'Connor RM, Grenham S, Dinan TG, Cryan JF. microRNAs as novel antidepressant targets: converging effects of ketamine and electroconvulsive shock therapy in the rat hippocampus. *Int J Neuropsychopharmacol* 2013: 1-8.
5. Zhao F, Qu Y, Zhu J, et al. miR-30d-5p Plays an Important Role in Autophagy and Apoptosis in Developing Rat Brains After Hypoxic-Ischemic Injury. *J Neuropathol Exp Neurol* 2017; **76**(8): 709-19.
6. Caserta S, Mengozzi M, Kern F, Newbury SF, Ghezzi P, Llewelyn MJ. Severity of Systemic Inflammatory Response Syndrome Affects the Blood Levels of Circulating Inflammatory-Relevant MicroRNAs. *Front Immunol* 2017; **8**: 1977.
7. Diez-Planelles C, Sanchez-Lozano P, Crespo MC, et al. Circulating microRNAs in Huntington's disease: Emerging mediators in metabolic impairment. *Pharmacol Res* 2016; **108**: 102-10.
8. Vargas-Medrano J, Yang B, Garza NT, Segura-Ulate I, Perez RG. Up-regulation of protective neuronal MicroRNAs by FTY720 and novel FTY720-derivatives. *Neurosci Lett* 2019; **690**: 178-80.
9. Jiang M, Wang H, Jin M, et al. Exosomes from MiR-30d-5p-ADSCs Reverse Acute Ischemic Stroke-Induced, Autophagy-Mediated Brain Injury by Promoting M2 Microglial/Macrophage Polarization. *Cell Physiol Biochem* 2018; **47**(2): 864-78.
10. Maffioletti E, Cattaneo A, Rosso G, et al. Peripheral whole blood microRNA alterations in major depression and bipolar disorder. *J Affect Disord* 2016; **200**: 250-8.
11. Satoh J, Kino Y, Niida S. MicroRNA-Seq Data Analysis Pipeline to Identify Blood Biomarkers for Alzheimer's Disease from Public Data. *Biomark Insights* 2015; **10**: 21-31.
12. Donzelli S, Mori F, Bellissimo T, et al. Epigenetic silencing of miR-145-5p contributes to brain metastasis. *Oncotarget* 2015; **6**(34): 35183-201.
13. Starhof C, Hejl AM, Heegaard NHH, et al. The biomarker potential of cell-free microRNA from cerebrospinal fluid in Parkinsonian Syndromes. *Mov Disord* 2019; **34**(2): 246-54.
14. Vaccaro TDS, Sorrentino JM, Salvador S, Veit T, Souza DO, de Almeida RF. Alterations in the MicroRNA of the Blood of Autism Spectrum Disorder Patients: Effects on Epigenetic Regulation and Potential Biomarkers. *Behav Sci (Basel)* 2018; **8**(8).

15. Schneider R, McKeever P, Kim T, et al. Downregulation of exosomal miR-204-5p and miR-632 as a biomarker for FTD: a GENFI study. *J Neurol Neurosurg Psychiatry* 2018; **89**(8): 851-8.

16. Xia Z, Liu F, Zhang J, Liu L. Decreased Expression of MiRNA-204-5p Contributes to Glioma Progression and Promotes Glioma Cell Growth, Migration and Invasion. *PLoS One* 2015; **10**(7): e0132399.

References for Supplementary Methods

17. Rojo DR, Prough DS, Boone DR, et al. Influence of stochastic gene expression on the cell survival rheostat after traumatic brain injury. *PLoS One* 2011; **6**(8): e23111.

18. Shimamura M, Garcia JM, Prough DS, Hellmich HL. Laser capture microdissection and analysis of amplified antisense RNA from distinct cell populations of the young and aged rat brain: effect of traumatic brain injury on hippocampal gene expression. *Mol Brain Res* 2004; **17**(1): 47-61.

19. Cherian L, Robertson CS, Goodman hJC. Secondary insults increase injury after controlled cortical impact in rats. *J Neurotrauma* 1996; **13**: 371-83.

20. Oliveros, J. C. (2015). Venny. An interactive tool for comparing lists with Venn's diagrams. http://bioinfogp.cnb.csic.es/tools/venny/index.html

21. Liu W, Wang X. Prediction of functional microRNA targets by integrative modeling of microRNA binding and target expression data. *Genome Biol* 2019; **20**(1): 18.

22. Kozomara A, Griffiths-Jones S. miRBase: annotating high confidence microRNAs using deep sequencing data. *Nucleic Acids Res* 2014; **42**(Database issue): D68-D73.

References for Table S1:

23. O'Connell RM, Taganov KD, Boldin MP, Cheng G, Baltimore D. MicroRNA-155 is induced during the macrophage inflammatory response. *Proc Natl Acad Sci U S A* 2007; **104**(5): 1604-9.

24. Tili E, Michaille JJ, Cimino A, et al. Modulation of miR-155 and miR-125b levels following lipopolysaccharide/TNF-alpha stimulation and their possible roles in regulating the response to endotoxin shock. *J Immunol* 2007; **179**(8): 5082-9.

25. Wang WX, Visavadiya NP, Pandya JD, Nelson PT, Sullivan PG, Springer JE. Mitochondria-associated microRNAs in rat hippocampus following traumatic brain injury. *Exp Neurol* 2015; **265**: 84-93.

26. Ying W, Tseng A, Chang RC, et al. MicroRNA-223 is a crucial mediator of PPARgamma-regulated alternative macrophage activation. *J Clin Invest* 2015; **125**(11): 4149-59.

27. de Gonzalo-Calvo D, Davalos A, Montero A, et al. Circulating inflammatory miRNA signature in response to different doses of aerobic exercise. *J Appl Physiol (1985)* 2015; **119**(2): 124-34.

28. Ge X, Han Z, Chen F, et al. MiR-21 alleviates secondary blood-brain barrier damage after traumatic brain injury in rats. *Brain Res* 2015; **1603**: 150-7.

29. Han Z, Chen F, Ge X, Tan J, Lei P, Zhang J. miR-21 alleviated apoptosis of cortical neurons through promoting PTEN-Akt signaling pathway in vitro after experimental traumatic brain injury. *Brain Res* 2014; **1582**: 12-20.

30. Sheedy FJ. Turning 21: Induction of miR-21 as a Key Switch in the Inflammatory Response. *Front Immunol* 2015; **6**: 19.

31. Wu D, Cerutti C, Lopez-Ramirez MA, et al. Brain endothelial miR-146a negatively modulates T-cell adhesion through repressing multiple targets to inhibit NF-kappaB activation. *J Cereb Blood Flow Metab* 2015; **35**(3): 412-23.

32. Iwasaki K, Yamamoto T, Inanaga Y, et al. MiR-142-5p and miR-486-5p as biomarkers for early detection of chronic antibody-mediated rejection in kidney transplantation. *Biomarkers* 2017; **22**(1): 45-54.

References for Supplemental Discussion

33. Lei P, Li Y, Chen X, Yang S, Zhang J. Microarray based analysis of microRNA expression in rat cerebral cortex after traumatic brain injury. *Brain Res* 2009; **1284**: 191-201.

34. Redell JB, Liu Y, Dash PK. Traumatic brain injury alters expression of hippocampal microRNAs: potential regulators of multiple pathophysiological processes. *J Neurosci Res* 2009; **87**(6): 1435-48.

35. Hu Z, Yu D, Almeida-Suhett C, et al. Expression of miRNAs and their cooperative regulation of the pathophysiology in traumatic brain injury. *PLoS One* 2012; **7**(6): e39357.

36. Meissner L, Gallozzi M, Balbi M, et al. Temporal Profile of MicroRNA Expression in Contused Cortex after Traumatic Brain Injury in Mice. *J Neurotrauma* 2016; **33**(8): 713-20.

37. Vuokila N, Lukasiuk K, Bot AM, et al. miR-124-3p is a chronic regulator of gene expression after brain injury. *Cell Mol Life Sci* 2018; **75**(24): 4557-81.

38. Balakathiresan N, Bhomia M, Chandran R, Chavko M, McCarron RM, Maheshwari RK. MicroRNA let-7i is a promising serum biomarker for blast-induced traumatic brain injury. *J Neurotrauma* 2012; **29**(7): 1379-87.

39. Sharma A, Chandran R, Barry ES, et al. Identification of serum microRNA signatures for diagnosis of mild traumatic brain injury in a closed head injury model. *PLoS One* 2014; **9**(11): e112019.

40. Bhomia M, Balakathiresan NS, Wang KK, Papa L, Maheshwari RK. A Panel of Serum MiRNA Biomarkers for the Diagnosis of Severe to Mild Traumatic Brain Injury in Humans. *Sci Rep* 2016; **6**: 28148.

41. Di Pietro V, Ragusa M, Davies D, et al. MicroRNAs as Novel Biomarkers for the Diagnosis and Prognosis of Mild and Severe Traumatic Brain Injury. *J Neurotrauma* 2017; **34**(11): 1948-56.

42. Di Pietro V, Porto E, Ragusa M, et al. Salivary MicroRNAs: Diagnostic Markers of Mild Traumatic Brain Injury in Contact-Sport. *Front Mol Neurosci* 2018; **11**: 290.

43. Redell JB, Moore AN, Ward NH, III, Hergenroeder GW, Dash PK. Human traumatic brain injury alters plasma microRNA levels. *J Neurotrauma* 2010; **27**(12): 2147-56.

44. Mitra B, Rau TF, Surendran N, et al. Plasma micro-RNA biomarkers for diagnosis and prognosis after traumatic brain injury: A pilot study. *J Clin Neurosci* 2017; **38**: 37-42.

45. Papa L, Slobounov SM, Breiter HC, et al. Elevations in MicroRNA Biomarkers in Serum Are Associated with Measures of Concussion, Neurocognitive Function, and Subconcussive Trauma over a Single National Collegiate Athletic Association Division I Season in Collegiate Football Players. *J Neurotrauma* 2019; **36**(8): 1343-51.

46. Campolettano ET, Gellner RA, Rowson S. High-magnitude head impact exposure in youth football. *J Neurosurg Pediatr* 2017; **20**(6): 604-12.

47. Bailes JE, Dashnaw ML, Petraglia AL, Turner RC. Cumulative effects of repetitive mild traumatic brain injury. *Prog Neurol Surg* 2014; **28**: 50-62.
